# Self-sorting and co-assembly control in multicomponent supramolecular hydrogels with dual monomer and polymer statistical distribution

**DOI:** 10.1038/s42004-025-01657-1

**Published:** 2025-08-28

**Authors:** Mary C. Jones, Leide P. Cavalcanti, Gregory N. Smith, Robert Dalgliesh, Stephen R. Euston, Filipe Vilela, Valeria. Arrighi, Gareth O. Lloyd

**Affiliations:** 1 School of Engineering and Physical Sciences, Heriot-Watt University, Edinburgh, EH14 4AS Scotland UK; 2 ISIS Neutron and Muon Source, Rutherford Appleton Laboratory, Science and Technology Facilities Council, Didcot, OX11 0QX Oxfordshire UK; 3 School of Chemistry, Joseph Black Laboratories, University of Lincoln, Lincoln, LN6 7TS UK

**Keywords:** Supramolecular polymers, Self-assembly

## Abstract

Multicomponent supramolecular polymer gels are a class of soft matter materials which form via the assembly of two or more small molecules. Different structures can be generated with interesting potential functions and applications. Insight into the assembly mechanism is key in the design of these systems for specific applications. Herein, a series of hydrogels with diverse structures and functionalities were synthesised. Using dynamic covalent chemistry as a key tool we show that it is possible to control the monomer assembly, forming both self-sorted and co-assembled polymers and gels from the same initial components. The hierarchical structure of the gels is difficult to elucidate. We emphasise the significance of small-angle neutron scattering (SANS) and spin-echo SANS (SESANS) measurements in characterising these intricate assemblies and demonstrate that these techniques are able to differentiate among self-sorted and co-assembled structures even when using chemically similar components.

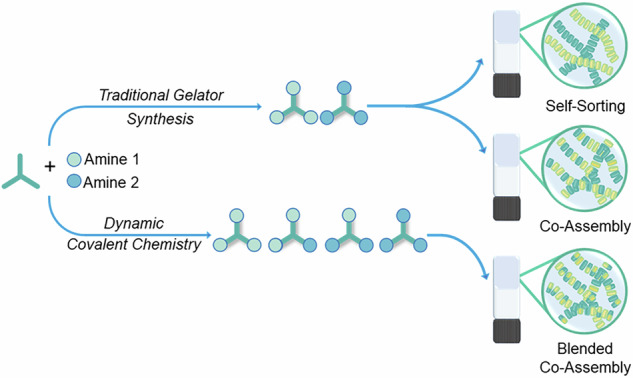

## Abstract (web summary)

Multicomponent supramolecular polymer gels can possess useful functions and applications, but the mechanisms of their assembly are not fully understood. Here, the authors use dynamic covalent chemistry as a tool to demonstrate that it is possible to control the monomer assembly, forming both self-sorted and co-assembled polymers and gels from the same initial components.

## Introduction

Supramolecular gels can form via the assembly of low molecular weight gelators (LMWGs), acting as monomers, through reversible non-covalent interactions into supramolecular polymers that form a self-assembled fibrillar network (SAFiN)^1,2^. The reversibility of the non-covalent interactions instils an enhanced tunability and, as a result, these systems have a large range of current and future applications spanning diverse fields such as organic electronics, drug delivery, and cell culture media, to name but a few^2–6^.

**Fig. 1:**
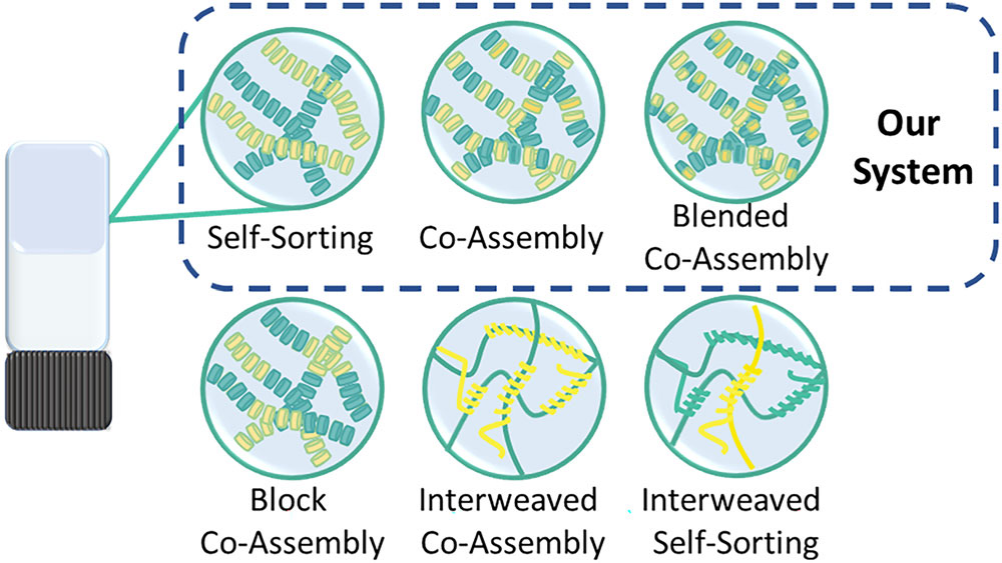
Assembly mechanisms in supramolecular gels. Schematic representation of some of the different supramolecular systems and gel networks formed from multicomponent assembly.

Supramolecular polymerisation of two or more different components can result in the formation of a multicomponent gel or polymer and allows fine-tuning of network properties such as the mechanical strength, spatio-temporal stability and electronic properties^2,7,8^. However, controlling the supramolecular co-polymerisation is not trivial. Different outcomes are possible, including narcissistic self-sorting, where each LMWG will form separate fibres; or at the opposite extreme, random co-assembly, where the LMWGs are distributed among the fibres in a non-ordered and unpredictable manner. There are, of course, several intermediate situations between these two extremes, such as block co-assembly or interweaved fibrils. (Fig. 1)^9,10^. Although predicting the assembly mechanism is difficult, by considering interaction energies between the building blocks, thermodynamic control over the assembly process can be established^6^. Self-sorted systems have been designed by selecting monomers with structural^9,11–15^/chiral mismatch^16^ and thermal annealing^17–19^ whereas co-assembly can be promoted by exploiting complementary electrostatics or introducing charge transfer interactions^9,20–28^. Many of these tools and ideas have been and will need to be utilised to develop out-of-equilibrium systems^27–34^.

One of the great challenges is the formation of both self-sorted and co-assembled supramolecular entities from the same building blocks, with very few examples previously reported^9,28,29^. Since assemblies are typically formed under thermodynamic control, by considering alternative kinetic pathways, it is possible to activate sequence control during assembly. A recent report on the use of chemical fuels and cycles by Singh, Hermans and co-workers shows how selecting monomers with different chemical reactivity can push a normally co-assembled system into a self-sorted set of assemblies^28^. Sequence-controlled supramolecular structures were also obtained by Sarkar et al. using kinetic pathways and seeding to direct their thermodynamically controlled systems to self-sort or form block co-polymers^9^. By astute selection of differing triggers for the induction of assembly, Adams and co-workers have shown that it is possible to switch between co-assembled (by anti-solvent) to self-sorted (kinetic pH change) systems^29^.

Here, we show that blending of the chemical functionality in multi-component systems makes it possible to switch between either a self-sorted pair of supramolecular gel networks or a co-assembled network. We utilise dynamic covalent chemistry to make multifunctional LMWGs and lock that dynamic functionality into specific patterns using a non-reversible tautomerisation, as previously demonstrated by our group^27^. This additional complexity then adds to the statistical assembly mechanism associated with the gel formation. The simple hypothesis for this work is that the more diverse and disordered the functional groups, the more likely the co-assembly of the monomers into a multicomponent supramolecular polymer. By contrast, a binary, less disordered, multicomponent mixture prefers to self-sort by sticking to familiar functionality, and it is thermodynamically less likely to co-associate.

Distinguishing between the different assembly mechanisms within gels is particularly challenging. Gels are constituted primarily of solvent (>95% w/w) and, therefore, common laboratory techniques, such as wide-angle X-ray diffraction (XRD) and microscopy, can provide useful insight but have limitations. This is because artefacts and/or structural changes may arise during sample preparation (e.g. drying)^35,36^. To circumvent these issues, non-destructive techniques such as small-angle neutron scattering (SANS) can be employed to probe the structure of the as-prepared, solvated samples^35,37^.

SANS measurements of the scattered intensity as a function of the scattering angle provide unique information on the shape and size of the scattering object^37,38^. SANS can also be used to investigate the sol-to-gel transition and/or to study the finer features of the gel networks of various systems^39–48^. However, as SANS is a less readily available technique, typically only feasible at large-scale facilities, it is unsurprising that it has not found widespread use in characterising supramolecular gels^49^.

The small LMWG molecules self-assemble into long fibres, which entangle and physically cross-link to form SAFiNs. Understanding the mechanism by which LMWGs pack into highly hierarchical structures requires the use of complementary techniques able to probe a very wide range of length scales. Recently, Adams and co-workers have championed the use of SANS to study the assembly mechanism of their multicomponent gels^50,51^. To look at longer length scales, ultra-small-angle neutron scattering (USANS) has been used^52,53^. Spin-echo small-angle neutron scattering (SESANS) can also be employed for this purpose^54,55^ but the technique has yet to be used to study supramolecular gel networks in detail.

Due to the structural similarity of the LMWGs used in this study, the elucidation of our assemblies was only possible by conducting SANS with complementary SESANS measurements. The work presented here can be used to elucidate design principles for controlling the supramolecular polymerisation, which can lead to programmable and information-rich materials.

## Results and discussion

### Gelator synthesis and selection

**Fig. 2:**
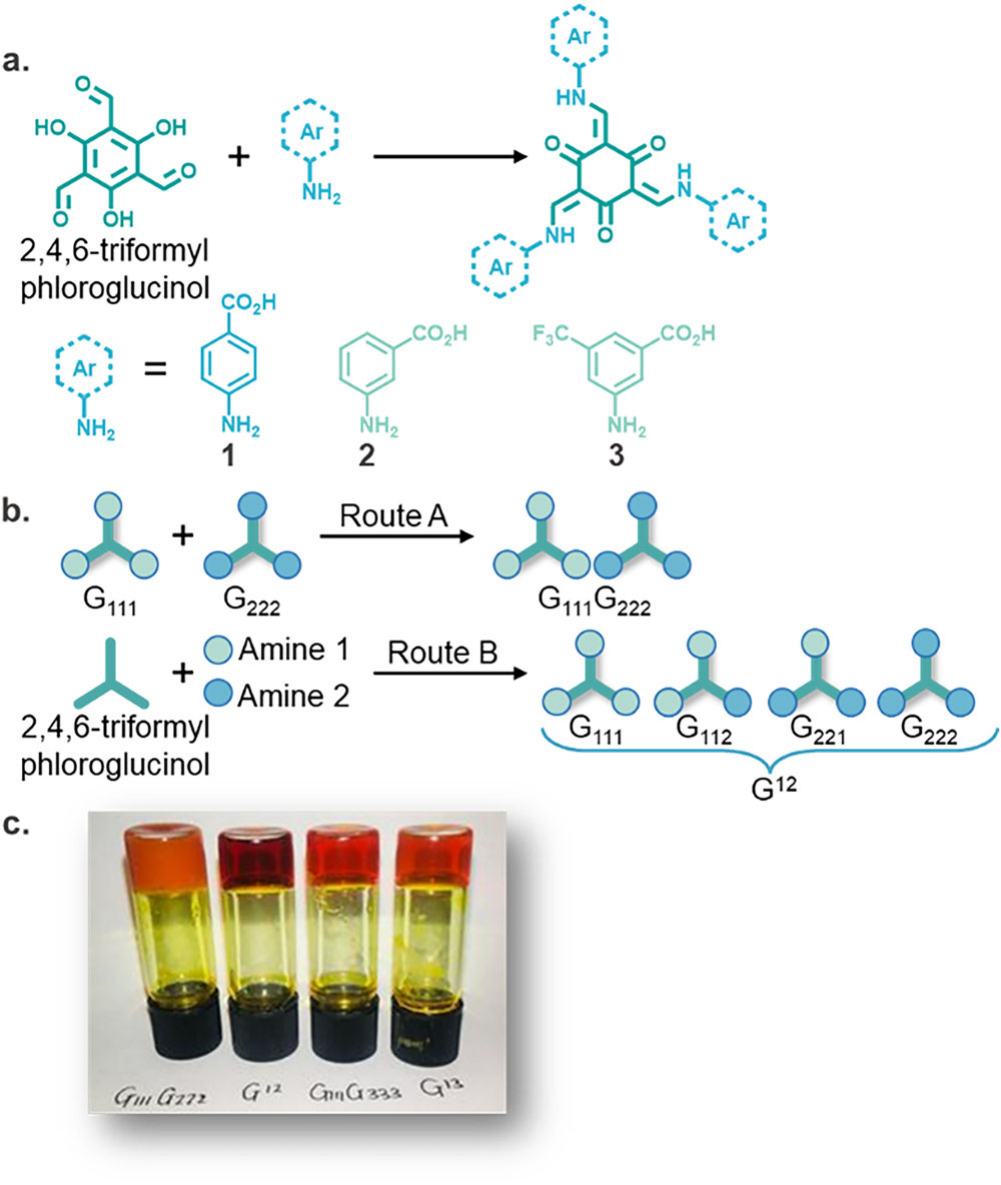
Chemical structure and characteristics of LMWGs and gels. **a** Synthesis of ketoenamine-based hydrogels. **b** Pictorial representation of the LWMGs formed by different gelation routes. **c** Multicomponent gels showing differences in transparency.

Three different building blocks were used to prepare LMWGs. Compounds **1** and **2** (Fig. 2a) are positional isomers of each other. Compound **3** contains the additional electron-withdrawing CF_3_ unit (Fig. 2a). Multicomponent tripodal ketoenamine-based hydrogels with a triformylphloroglucinol core can be synthesised by two distinct routes^27,56^. Route A involves the mixing of two ‘pre-formed’ LMWGs (e.g. **G**_**111**_ with **G**_**222**_), whereas route B describes the reaction of two different aminobenzoic acids with 2,4,6-triformylphloroglucinol (Fig. 2a) to generate a four-component mixture (e.g. a gelator mixture **G**^12^ would be **G**_**111**_, **G**_**112**_, **G**_**122**_, and **G**_**222**_ in equal quantities). The aminobenzoic acid components were sourced commercially, while the synthesis of 2,4,6-triformylphloroglucinol has been widely published^57–61^. Syntheses and characterisation data are available in the “Synthesis and Characterisation” section of the SI. In route B (Fig. 2b) all starting materials (2,4,6-triformylphloroglucinol and two aminobenzoic acids) are reacted together simultaneously prior to gelation. The first step of the reaction consists of the formation of imine groups. Dynamic covalent chemistry^62^ operates during the formation of the imine intermediates and, provided sufficient time is allowed for equilibrium to be reached, an equal quantity of four gelators (Fig. 2b) is formed. As shown elsewhere^27^ the dynamic covalent chemical distribution for the reaction is 1 : 1 : 1 : 1 from HPLC analysis. This equal ratio of the LMWGs is subsequently locked in place by the tautomerisation^27,56^.

Gelation is triggered by dissolving the LMWGs at high pH (NaOH/H_2_O, above the apparent p*K*_*a*_ value of all starting materials and products) followed by the addition of glucono-δ-lactone (GdL). GdL acts as a slow release of protons and thus lowers the overall pH of the system below the apparent p*K*_*a*_ of the LMWGs, reducing their solubility and resulting in the formation of a solid network (Table S1)^63^. In our previous work^27,56^, we reported the synthesis of the two gelator mixes **G**_**111**_**G**_**333**_ and **G**^**13**^, which, as shown in that work, are effectively 1:1 and 1:1:1:1 in terms of components, respectively. These gels are visually indistinguishable (Fig. 2c), and using literature-supported characterisation in the form of XRD, pH titrations to determine variation in the apparent p*K*_a_ values, and solution ^1^H NMR, the two gelator mixes appeared to form a co-assembled fibrous network. This co-assembly occurs even though the apparent p*K*_a_ values of **G**_**111**_ and **G**_**333**_ are different enough to expect self-sorting during the gradual lowering of the pH, as has been seen in the work by Adams and co-workers^7,12,13^. Dissimilar to the **G**_**111**_**G**_**333**_ and **G**^**13**^ gelator mixes, the new multicomponent gels **G**_**111**_**G**_**222**_ and **G**^**12**^ are visually different, as shown in Fig. 2c where **G**_**111**_**G**_**222**_ is opaque, while **G**^**12**^ is transparent. We thus aimed to determine if different assembly mechanisms were occurring and, consequently, if two distinct gel networks were formed. Compounds **1** and **2** are positional isomers of each other; thus, the apparent p*K*_a_ values of **G**_**111**_ and **G**_**222**_ are nearly identical (6.1–5.8 and 6.5–5.9, respectively). Due to this similarity, any analytical technique following the kinetics of formation would not be able to resolve what type of assembly was occurring, self-sorted or co-assembled. As expected, the use of NMR for following the kinetics of assembly provided no notable variation (Fig. S7) while the XRD for single-component **G**_**111**_, **G**_**222**_ and multicomponent **G**_**111**_**G**_**222**_ and **G**^**12**^ are very similar. (Figs. S8 to S10) In previous work and as part of this study^27,56^ we viewed similar gels using microscopy (SEM, AFM, TEM, and super-resolution microscopy), but the resolution offered by these techniques was found to be insufficient for comparing the different gel networks. We have attempted to re-image the new gels with no further improvements in image quality; thus, we have not presented images for the materials in this paper. To overcome these limitations, we turned to neutron scattering techniques. As discussed below, SANS and SESANS offer unique tools able to distinguish between different assemblies and networks, including co-assembly or self-sorting processes.

### Single-component gels: determination of assembly model

**G**_**111**_, **G**_**222**_ and **G**_**333**_ all form single-component hydrogels. We have described previously the chemical and gel properties of **G**_**111**_ and **G**_**333**_ and full characterisation of **G**_**222**_ is reported in the supplementary information^27,56^.

The LMWG monomers are discotic molecules. Molecular dynamic simulations (see “Molecular Dynamics Simulations” in SI for detail) show that they form one-dimensional face-to-face stacks stabilised by *π*-*π* interactions and intramolecular hydrogen bonding allowing for a rigid structure, reminiscent of their liquid crystal and covalent organic framework counterparts^64–67^.

Despite their chemical similarities, single-component gels of **G**_**111**_ and **G**_**222**_ are strikingly different in appearance, with the latter forming a fully transparent gel. A difference in transparency is typically a result of the width of gel fibres and the extent of aggregation within the system^1–5,7,8^. As discussed later, through model fitting, SANS provides a measure of a range of structural parameters of the fibres within the gel networks and therefore allows us to probe the underlying reasons for the differences in optical transparency without drying the gel samples^35,41^.

**Fig. 3:**
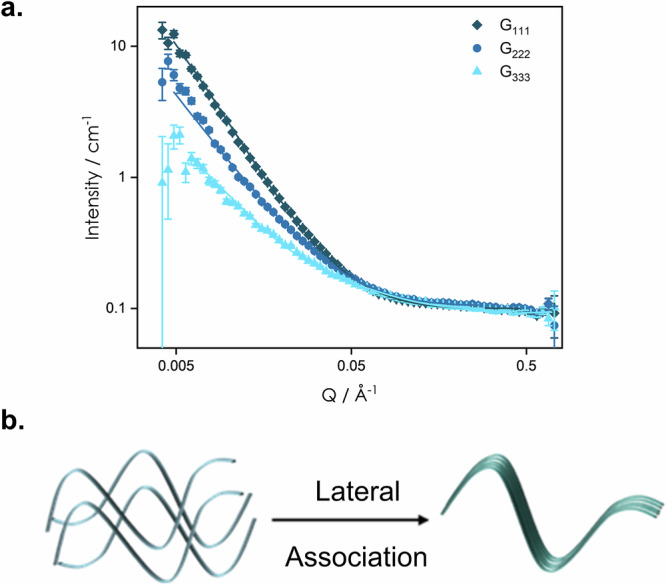
Comparison between SANS data of pure gels. **a** Scattering curves for pure gels **G**_**111**_, **G**_**222**_ and **G**_**333**_ (symbols) with a fit to a flexible elliptical cylinder model (lines). **b** Schematic showing lateral association of fibres resulting in an elliptical cross-section and decreased flexibility of the fibres.

The SANS data of the pure gels are plotted in Fig. 3a. These samples were measured at a fixed concentration (1 wt%) in D_2_O to provide contrast between the solid network and aqueous solution. The notable difference between the scattering profiles indicates a difference in the structure of the primary fibres (Fig. 3).

The scattering vector, *Q*, is inversely proportional to the size of the scattering objects. Thus, the absence of a plateau at the lowest experimental *Q* values (i.e. the absence of a detectable, shape-independent Guinier region) suggests that the length of our fibres lies outside the size accessible (d = 2π/*Q* < 151 nm) with the probed *Q* range. As a result, the scattering patterns will be most representative of changes to the fibril diameters.

A *Q* dependency of -1 is expected for cylindrical rod-like scatterers and is seen frequently in the literature for supramolecular gels^44,68^ and benzene-1,3,5-tricarboxamide-based supramolecular polymers, which have a similar C_3_ symmetric, discotic shape as our LMWGs^69–71^. However, fitting our data to a cylinder or a flexible cylinder model proved unsuccessful. To determine the *Q* dependence, the SANS data were fitted to a power law:

1 $$I\left(Q\right)={I(0)\times Q}^{-\alpha }+B$$

where *I*(0) is the scale, *α* characterises the *Q* dependence and *B* represents the background. Using Eq. (1), Q dependencies of -2.1, -1.8 and -1.6 were determined for gels **G**_**111**_ to **G**_**333**_, respectively. Table 1 presents a selection of parameters determined from fits of all the SANS samples measured. Full sets of fitting parameters are listed within the SI (Tables S3 and S4). A *Q* dependency closer to −2 (*α* = 2) suggests the presence of ribbon-like fibres, giving a lamellar-type structure (Fig. 3b). This is in line with the XRD data collected from dried samples (Figs. S8 to S10) in which peak positions of Bragg-like reflections have ratios of 1,2,3,4, which is consistent with lamellar-type packing^72^. From the *Q* dependency and comparison with similar systems in literature, a flexible elliptical cylinder model^71–74^ was chosen and fitted to the SANS data of the gels to extract dimensional information (Fig. 3, a detailed description of this process is provided in the “Small-Angle Neutron Scattering (SANS)” section of the SI)^48,75,76^. The elliptical cross-section is likely to arise from the lateral association of fibres (Fig. 3b)^48,75,76^.

**Table 1:** Selected parameters from fits using the power law (Eq. 1) and the flexible elliptical cylinder^77^ models for all measured gels

Gel	*α*^a^	Radius /Å	Axis ratio	Kuhn length /Å
**G**_**111**_	2.125 ± 0.006	8.5 ± 0.1	14 ± 2	281 ± 41
**G**_**222**_	1.788 ± 0.008	7.4 ± 0.4	2.80 ± 0.01	40 ± 5
**G**_**333**_	1.38 ± 0.01	8.50 ± 0.03	2.4 ± 0.1	146 ± 6
**G**_**111**_**G**_**222**_	2.241 ± 0.006	7.9 ± 0.5	12 ± 2	144 ± 8
**G**^**12**^	1.752 ± 0.009	7.60 ± 0.02	2.5 ± 0.2	45 ± 6
**G**_**111**_**G**_**333**_	1.544 ± 0.008	8.5 ± 0.3	3.1 ± 0.1	97 ± 4
**G**^**13**^	1.64 ± 0.01	8.47 ± 0.03	2.5 ± 0.1	88 ± 3

^a^Power law exponent, *α*, was determined by fitting the SANS data to Eq. (1). Fits and the full set of fitting parameters are given in the SI.

The flexible cylinder elliptical model in SasView^77^, used to fit the SANS data, is a function of the following structural parameters: (1) the radius of the cylinder, (2) the axis ratio, i.e. the major radius divided by the minor radius, (3) the Kuhn length characterising the flexibility of the cylinder and (4) the contour length. The minor radii for the three gels were found to vary between 7.4 and 8.5 Å, which is approximately the size of one molecule, as confirmed through molecular dynamics simulations (see “Molecular Dynamics Simulations” in SI for details). Hence, the larger the axis ratio, the greater the degree of lateral association and the more elliptical the cross-section. Values of axis ratios in Table 1 follow the same trend as *α* from Eq. 1, and so the two models (Eq. 1 and the elliptical model) indicate that the greatest extent of lateral association occurs for **G**_**111**_, which is in agreement with the opaque appearance of the gel.

The Kuhn length parameter gives an indication of the flexibility of the fibres. The larger the difference between the Kuhn and the cylinder lengths, the more flexible the fibres. For pure and multicomponent gels of **G**_**111**_ and **G**_**222**_ the contour length was fixed at the same value and therefore the smaller Kuhn lengths of **G**_**222**_ and **G**^**12**^ indicate more flexible fibres.

**Fig. 4:**
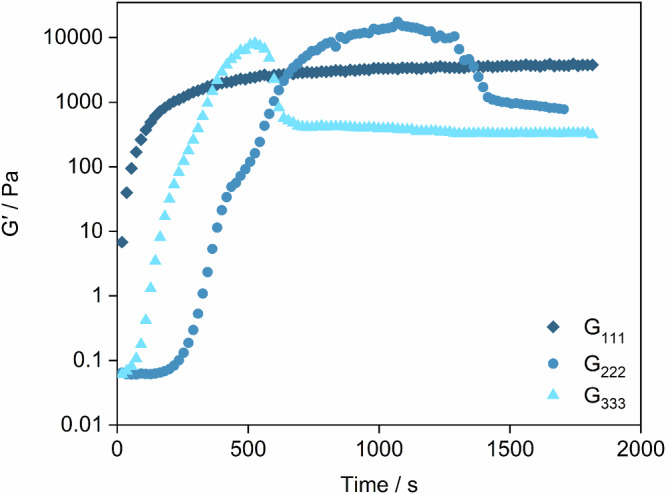
Time sweep measurements for pure gels. Time sweeps for **G**_**111**_, **G**_**222**_ and **G**_**333**_ showing the initial robustness of the **G**_**222**_ and **G**_**333**_ before syneresis occurs.

Time, frequency, and amplitude sweep rheological measurements were carried out under the same conditions as the SANS data to confirm the smaller mesh size (which normally arises in a more robust gelatinous material)^1–5,7,8^. As SANS measurements were carried out in D_2_O, the effect of switching from H_2_O to D_2_O was also investigated, and it was found that the rate of gelation decreased. This is unsurprising as the rate of GdL hydrolysis is reduced in a deuterated solvent^78^. Regardless, overall trends are consistent and therefore complete rheological measurements were carried out in H_2_O. The time sweeps (Fig. 4) show that **G**_**222**_ and **G**_**333**_ initially form mechanically stronger gels than **G**_**111,**_ confirming the hypothesis that they form more compact, interlocked network structures. However, once the maximum strength is reached, a decrease in G^′^ values is observed, followed by a lower plateau at longer time sweeps. This behaviour is indicative of syneresis, i.e. shrinking of the gel as water is expelled. Lateral association of fibres is known to affect the mechanical strength of a gel and its stability. We believe that the absence of syneresis observed for **G**_**111**_ (Fig. 4) is consistent with the larger extent of lateral association and greater stiffness of this gel, as indicated by the SANS measurements (Table 1).

Due to the syneresis, limited information can be extracted from the frequency or amplitude sweeps; however, all are consistent with the expected behaviour of gel materials (Figs. S24 to S26).

SESANS was used to further investigate the network structures of **G**_**111**_, **G**_**222**_ and their multicomponent gels. SESANS is an emerging technique that, in contrast to the SANS measurements obtained in reciprocal (Q) space, measures the extent of the depolarisation of a spin-polarised beam as a function of a real-space parameter, the spin-echo length. The spin-echo length can be defined as the correlation distance probed in the sample. For a detailed explanation of the SESANS technique, we refer readers to the referenced articles^54,79^.

**Fig. 5:**
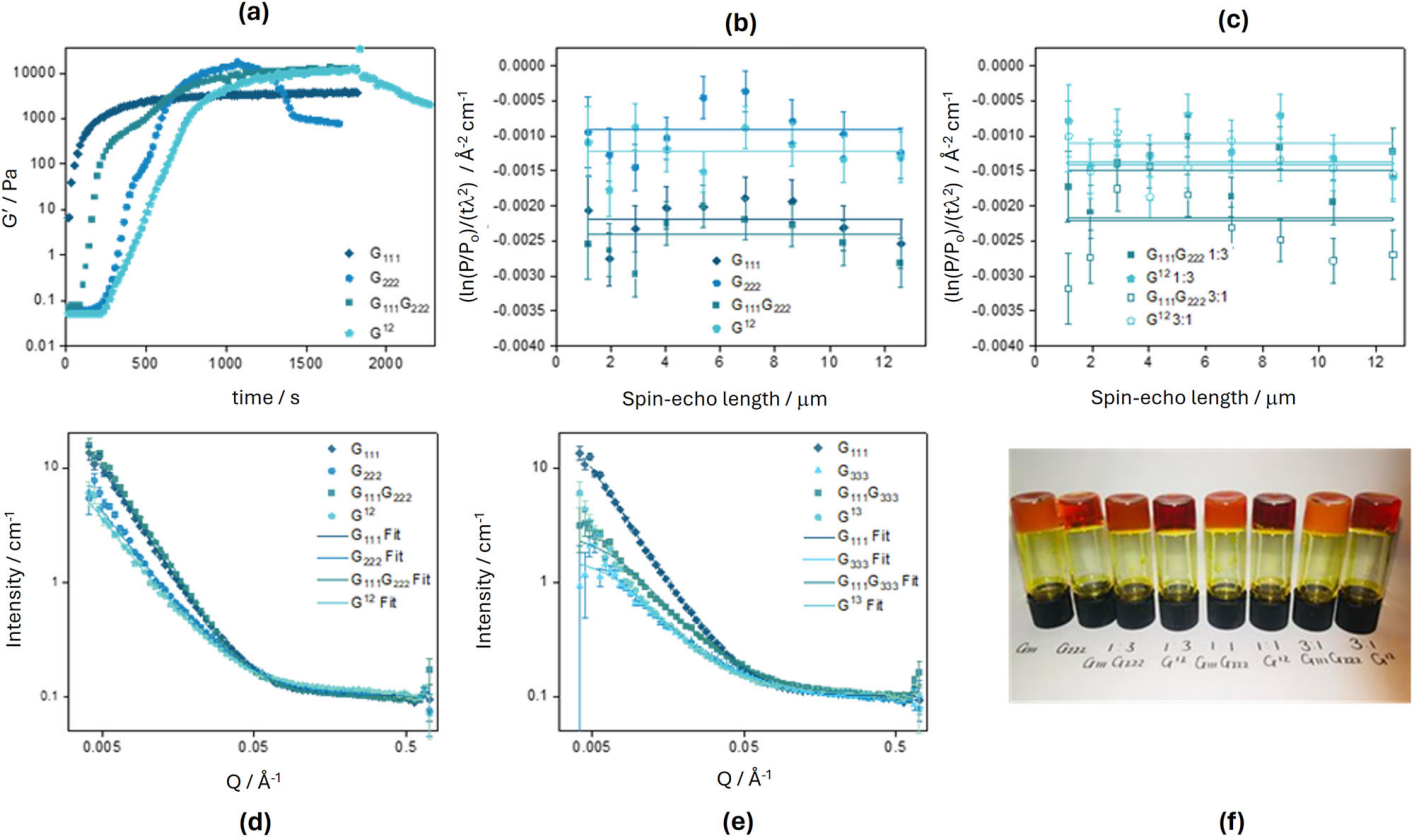
Structural and rheological properties of pure and multicomponent gels. **a** Time sweeps for pure gels **G**_**111**_, **G**_**222**_ and their **G**_**111**_**G**_**222**_ and **G**^**12**^ multicomponent gels. **b** SESANS data for **G**_**111**_, **G**_**222**_ and their **G**_**111**_**G**_**222**_ and **G**^**12**^ multicomponent gels. The lines are guides to the eye. **c** SESANS data for the 1:3 and 3:1 **G**_**111**_**G**_**222**_ and **G**^**12**^ multicomponent gels showing the general shift towards more **G**_**111**_ or **G**_**222**_ characteristics. **d** Scattering curves for pure gels **G**_**111**_, **G**_**222**_ and their **G**_**111**_**G**_**222**_ and **G**^**12**^ multicomponent gels with fit to an elliptical cylinder model. **e** Scattering curves for pure gels **G**_**111**_, **G**_**333**_ and their **G**_**111**_**G**_**333**_ and **G**^**13**^ multicomponent gels with fit to an elliptical cylinder model. **f** Image of the mixed ratios gels of **G**_**111**_, **G**_**222**_ and their **G**_**111**_**G**_**222**_ and **G**^**12**^ multicomponent versions, with the transparency indicating how the gels are altering.

The SESANS data in Fig. 5 display the normalised SESANS signal, ln(*P*/*P*_0_)/(*t*λ^2^), where *P* is the polarisation of the beam through the sample, *P*_0_ is the polarisation of the reference beam going through the empty instrument, *t* is the sample thickness, and *λ* is the wavelength. The depolarisation depends on *t* and *λ*, so this normalised parameter removes instrumental dependence. As shown in Fig. 5c, e the normalised SESANS signal from our samples is constant as a function of spin-echo length, which indicates that there are no structural features at the length scales probed (between approximately 0.9 and 13.8 μm) and that the samples appear homogeneous. We note that the depolarisation effect varies from sample to sample (Fig. 5).

The normalised SESANS signal, ln(*P*/*P*_0_)/(*tλ*^2^), is related to the contrast, the volume fraction and the correlation length (see “Spin-Echo Small-Angle Neutron Scattering (SESANS)” section in SI for details). Due to the similar structures of our LMWGs, the contrast is the same for all samples. By doing measurements at the same concentration, we can assume a constant volume fraction. Thus, all parameters related to the loss in polarisation are identical, except for the correlation length^54^. Therefore, by measuring the extent of depolarisation, we can directly compare the correlation lengths of our samples.

We note that to increase the SESANS signal, the SESANS samples were five times more concentrated (5 wt%) than those used for the SANS experiments (1 wt%). Additional SANS measurements were run on the pure gels **G**_**111**_ and **G**_**222**_ at the higher concentration, in a thicker cell, to mimic the SESANS conditions (Fig. S18). In the absence of structural changes, the scattered intensity should be directly proportional to the concentration of the scattering objects. As shown in Figs. S18 to S20, this is not the case for our gels. A network structure forms, with increasing concentration and therefore the scattering data provide a measure of the network mesh size.

**G**_**111**_ was found to depolarise the beam to a greater extent than **G**_**222**_, indicating a larger mesh size (Fig. 5c). The Kuhn length extracted from the SANS data (Table 1) indicates that **G**_**222**_ is much more flexible than **G**_**111**_. Therefore, the shorter correlation length associated with the SESANS depolarisation of **G**_**222**_ is unsurprising given that the thinner and more flexible fibres of these gels would have a greater propensity to form entanglements^1–5,7,8^. This is fully supported by SANS measurements at 5 wt% (Fig. S20), which are well described by a correlation length model (see section 6.4 of the SI).

Overall, it can be hypothesised that the greater propensity for lateral association, as seen for **G**_**111**_, is related to the substitution pattern on the aromatic amines, with the para-substituted amines producing the most elliptical and least flexible fibres which in turn produce mechanically weaker gel networks.

Thus, the combined SANS and SESANS data provide insight into the hierarchical structure of the gel networks. The initial columnar aggregates, confirmed by XRD analysis and molecular dynamics simulations, undergo lateral association into elliptical fibres, as shown by fitting the SANS data to a flexible elliptical cylinder model. From the SANS fitting parameters, we can estimate the degree of association (see “Determination of the Degree of Association” section in the SI). A large degree of association (12–15 strands) limits the flexibility of the fibres, evident from the larger Kuhn length, and consequently, the SAFiN mesh size, resulting in opaque gels. Conversely, transparent gels have a limited aggregation of approximately 2-3 strands forming a larger number of fibre,s which are more flexible (smaller Kuhn length) and thus a denser and mechanically stronger gel network is formed. It can be hypothesised that the greater propensity for lateral association, as seen for **G**_**111**_, is related to the substitution pattern on the aromatic amines, with para-substituted amines exhibiting the most elliptical fibril cross sections.

With these clear assembly models for the pure gels in hand, we can now turn to the data and models for the multicomponent gels.

### Multicomponent gels: self-sorted and/or co-assembly

Compounds **1** and **2** are regioisomers and consequently **G**_**111**_, **G**_**222**_, **G**_**111**_**G**_**222**_ and **G**^**12**^ exhibit near identical apparent p*K*_a_ values. As a result, pH titrations and ^1^H NMR could not be used to distinguish between different assembly mechanisms, as was the case for **G**_**111**_**G**_**333**_ and **G**^**13**^^27,56^. As the apparent p*K*_a_ values are nearly identical, it is highly unlikely that the kinetic behaviour supplied by the judicious use of GdL, as seen by others^29^, can contribute to the self-sorting of the different gelators. So this study aims to answer the question: Do compounds **1** and **2** match the assembly of **1** and **3**? The XRD patterns for the xerogels (Figs. S8 to S10) exhibit only minor differences, and no conclusion can be drawn with regard to the assembly mechanism.

In multicomponent gels, the two LMWGs may work synergistically to improve the mechanical properties or antagonistically, producing a weaker gel than the individual LMWGs^35^. Time sweeps during gel formation showed that the maximum value for the elastic modulus (G′) is higher for the multicomponent gels than for **G**_**111**_ (Fig. 5a). It can therefore be concluded that the presence of **G**_**222**_ strengthens both multicomponent gels. Similar to **G**_**222**_, the **G**^12^ gel exhibits syneresis, albeit delayed with respect to the pure gel (Fig. 5a). Overall, different gel properties are formed *via* the different synthesis routes, but it is unclear if this is the product of different assembly mechanisms or the chemical differences arising from the dynamic covalent chemistry giving mechanically different but structurally similar networks.

To fully characterise our gels, SANS measurements were conducted, and prior to a detailed analysis, it is immediately obvious that the scattering curves of the **G**_**111**_**G**_**222**_ and **G**^12^ multicomponent gels are significantly different (Fig. 5d). Again, power law and flexible elliptical cylinder models were fitted to the data, and values of fitting parameters are reported in Table 1.

Further details regarding the fitting procedure can be found in sections 6.2 and 6.3 of the SI. As shown in Table 1, all fitting parameters for the **G**_**111**_**G**_**222**_ gel are closer to those of **G**_**111**_ compared to **G**_**222**_, with a significant amount of lateral association represented by the relatively large axis ratio and the *Q*^-2.2^ dependence. Conversely, the values extracted for the **G**^**12**^ gel are closer to those of **G**_**222**_. The same trend is observed for SESANS measurements (Fig. 5c), with both the **G**_**111**_ and **G**_**111**_**G**_**222**_ gels depolarising the beam to a greater extent than both the **G**^**12**^ and **G**_**222**_ gels. This trend substantiates longer correlation lengths for the **G**_**111**_ and **G**_**111**_**G**_**222**_ gels, resulting from limited entanglements due to the thicker, less flexible (based on the Kuhn length values) and less abundance of the fibres.

In a self-sorting system, the scattering would be expected to be more representative of **G**_**111**_ as its fibres are larger and would therefore dominate the scattering, as is the case for the **G**_**111**_**G**_**222**_ gels. However, the scattering profile for the **G**^**12**^ gel resembles closer that of **G**_**222**_ which suggests a co-assembly mechanism is in play and with interdigitating LMWGs, the lateral association typically seen for **G**_**111**_ is limited.

As mentioned previously, a common approach for designing self-sorting supramolecular polymers and gels consists of combining substantially different compounds^9,11–15^. Therefore, with LMWGs 1 and 2 being regioisomers, it is unsurprising that **G**^**12**^ would form a co-assembled gel network. However, by changing the synthesis route, unprecedented behaviour arises and a self-sorting mechanism is accessed from the same starting materials for **G**_**111**_**G**_**222**_. It is believed that this propensity for homo-recognition despite similar molecular structures is a result of the differing substitution pattern on the peripheral leg units, resulting in the formation of LMWGs with slightly different shapes^10^.

For comparison purposes and to further corroborate the co-assembling mechanism previously suggested for both **G**_**111**_**G**_**333**_ and **G**^**13**^ gels^27^, SANS experiments were carried out. As **G**_**111**_ and **G**_**333**_ have distinctly different apparent p*K*_a_ values, we can highlight that the kinetics of the pH change during the gel setting appear not to influence the selection to co-assembly and indicate that the intermittency of the components is likely occurring before gelation^27^. A different scenario than for **G**_**111**_**G**_**222**_ and **G**^**12**^ emerges, where the scattering curves for the **G**_**111**_**G**_**333**_ and **G**^**13**^ gels are similar (Fig. 5e). This is consistent with both gels co-assembling. As was the case for the **G**^**12**^ gel, which is also thought to co-assemble, the lateral association is limited for both gels, with the elliptical cylinder model returning a small axis ratio and the power law model returning a lower Q dependency in comparison to the **G**_**111**_ and **G**_**111**_**G**_**222**_ gels (Table 1).

Therefore, counterintuitively, despite the substantial structural mismatch and differing apparent p*K*_a_ values of the LMWGs, **G**_**111**_**G**_**333**_ and **G**^**13**^ form co-assembling SAFiNs *via* both synthetic mixing methods. However, co-assembly can be explained and triggered by the formation of donor-acceptor charge transfer-like π-π interactions^20–25^. MD simulations and literature highlight the *π*-*π* propensity of the monomers^64–67^. Due to the highly electron-withdrawing nature of the CF_3_ group, the aromatic rings of the **3** components will be electron-deficient with respect to the **1** components, and it is likely that ”aromatic donor-acceptor” interactions encourage co-assembly^80^. For **1** and **2**-based compounds such charge transfer-like π-π interactions are not possible, and despite the similar structures, the different substitution pattern is sufficient to encourage self-recognition.

The switch to the co-assembly mechanism during **G**^**12**^ gelation (in contrast to the self-sorting of **G**_**111**_**G**_**222**_) is therefore a consequence of the presence of asymmetric LMWGs formed *via* the dynamic covalent chemistry and the blending of the monomers, which encourages diversity and discourages self-recognition

Having confirmed the switch in the assembly mechanism by changing the synthetic route for a 1:1 ratio mixture for **G**_**111**_**G**_**222**_ and **G**^**12**^, we used SESANS (Fig. 5c) and rheology (Fig. S30) to investigate the effect of varying the ratio of the components. As shown in Fig. 5f, the three **G**_**111**_**G**_**222**_ gels are visually opaque compared to their **G**^**12**^ counterparts, suggesting similar assembly mechanisms. For a 3:1 ratio, the difference between **G**_**111**_**G**_**222**_ and **G**^**12**^ gels is still evident (Fig. 5c). In the case of a co-assembling mechanism, mixed fibres are produced. and the presence of **G**_**222**_ prevents the greater degree of lateral association and longer correlation length seen for pure fibres of **G**_**111**_. Therefore, the lower depolarisation for the **G**^**12**^ gel is indicative of possible co-assembly and even with a higher ratio of **G**_**111**_ in the gel, the small amount of **G**_**222**_ in the mixed fibres still limits the association and thus, the correlation length. The **G**_**111**_**G**_**222**_ gel depolarises the beam to a greater extent, suggesting that a self-sorting mechanism is occurring and that the larger fibres and longer correlation lengths of **G**_**111**_ dominate the scattering as seen with the 1:1 ratio. From the rheology, it was found that the syneresis, previously observed for the **G**^**12**^ (1:1) gel, does not occur for the higher ratio of **1** (**G**^**12**^ 3:1), and the gels are still different, with the **G**_**111**_**G**_**222**_ 3:1 gel forming a tighter gel network. (Fig. S30) When the ratio is reversed, the situation is less clear. The extent of depolarisation for the **G**_**111**_**G**_**222**_ 1:3 gel is no longer much greater than for **G**_**222**_ and the **G**^**12**^ 1:3 gel. (Fig. 5c) This is to be expected due to the slightly more transparent appearance of the gel and could be indicative of a change in assembly from self-sorting to co-assembly. However, with only 25% **G**_**111**_ present, even in the case of self-sorting, there would be a reduced number of the larger fibres of **G**_**111,**_ and this could account for the smaller correlation length, decreasing the depolarisation.

## Conclusion

In this work, we have shown how a blending method can be used to overcome self-recognition of monomers within a supramolecular polymer, allowing for a switch from a self-sorted set of networks into a co-assembled network. A four-component mixture was synthesised using dynamic covalent chemistry, and the properties of the resulting gel (**G**^**12**^) were compared to the two-component gel mixture (**G**_**111**_**G**_**222**_). By using SANS (primarily) with complementary SESANS, MD, XRD, and rheological data, we have been able to establish the assembly mechanism responsible for forming the gel fibres. Properties of **G**^**12**^ and **G**_**111**_**G**_**222**_ are compared to those of previously reported gels (**G**_**111**_ and **G**_**333**_**)**^27^. We have shown that, depending on the strength of the *π*−*π* interactions between the different LMWGs, it is possible to form either self-sorted or co-assembled gels. The addition of an electron-withdrawing CF_3_ moiety leads to an electron-deficient *π*-system, which tends to co-assemble. This effect appears to dominate even though the differing apparent p*K*_a_ values of the LMWGs should result in kinetic separation of the assembly through the use of the GdL. On the contrary, when using two structurally similar LMWGs with almost identical apparent p*K*_a_s, **1** and **2**, we find that homo-recognition is the preferred route, allowing self-sorted gels to be formed. By exploiting dynamic covalent chemistry, asymmetric LMWGs were formed. In this case, a co-assembled gel is obtained, thus providing a facile route for producing different gels for specific applications from the same components (**1** and **2**). We aim to use this technology to develop active components embedded into the gel’s fibres either in their self-sorted or co-assembled network, and we hope that the concept of blending supramolecular monomers can be applied to select between self-sorted or co-assembled supramolecular polymers with other potentially multifunctional monomers.

## Methods

### Materials

See Section 1.1 of Supplementary Information (SI).

### Experimental techniques and MD simulations

See Sections 1.2 to 1.10 of SI.

### Synthetic procedures

See Sections 2.1 to 2.5 and Figs. S1, S2, S3 and S5 of SI.

### Characterisation of LMWGs

See Sections 2.1 to 2.4, Figs. S4 and S6.

### Sample preparation

See Sections 3 and 4 of SI.

### Supplementary Information

The Supplementary Information file provides details on the methods used in this work and data analysis.

## Supplementary information

Supplementary Material

## Data Availability

The SANS2D experiment at ISIS was allocated beam time under experiment number RB2010573 and the SESANS on LARMOR was assigned the experiment number RB2010583.
