# Supplementary material for: Self-sorting and co-assembly control in multicomponent supramolecular hydrogels with dual monomer and polymer statistical distribution
Source: Commun Chem. 2025 Aug 28;8:265. doi: 10.1038/s42004-025-01657-1 (PMC12394493; doi:10.1038/s42004-025-01657-1)
Supplement: Supplementary file 1 — Supplementary Material [file 42004_2025_1657_MOESM1_ESM.pdf]

Supporting Information

Self-Sorting and Co-Assembly Control in Multicomponent Supramolecular Hydrogels with Dual Monomer and Polymer Statistical Distribution

Mary C. Jones†, Leide P. Cavalcanti⊥, Gregory N. Smith⊥, Robert Dalgliesh⊥, Stephen R. Euston†, Filipe Vilela†*, Valeria Arrighi†*, Gareth O. Lloyd‖*

† School of Engineering and Physical Sciences, Heriot-Watt University, Edinburgh, Scotland, United Kingdom, EH14 4AS

⊥ ISIS Neutron and Muon Source, Rutherford Appleton Laboratory, Science and Technology Facilities Council, Didcot, Oxfordshire, United Kingdom, OX11 0QX

‖School of Chemistry, Joseph Black Laboratories, University of Lincoln, Lincoln, United Kingdom, LN6 7TS

Corresponding email: glloyd@lincoln.ac.uk, v.arrighi@hw.ac.uk, f.vilela@hw.ac.uk.

Contents

1.0. Experimantal Details 4
1.1 Chemical List 4
1.2 NMR 4
1.3 IR 4
1.4 Mass Spectrometry 4
1.5 pH Meter 4
1.6 X-Ray Diffraction (XRD) and Scattering 4
1.7 Rheology 4
1.8 Molecular Dynamic (MD) Simulations 4
1.9 Small-Angle Neutron Scattering (SANS) 5
1.10 Spin-Echo SANS (SESANS) 5
2.0 Synthesis and Characterisation 6
2.1 2,4,6-Triformylphlorogluciol 6
2.2 G_{111} (conformers C_3 and C_s 3:1) 6
2.3 G_{222} (conformers C_3 and C_s 3:1) 6
2.4 G_{333} (conformers C_3 and C_s 3:1) 7
2.5 pK_a Values 8
2.5.1 pH Titration for G_{222} 8
3.0 Hydrogel Preparation 9
3.1 Single Component Gels (G_{111}, G_{222} and G_{333}) 9
3.2 Multicomponent Gels Route A ($G_{111}G_{222}$, and $G_{111}G_{333}$) 9
3.3 Multicomponent Gels Route B (G^{12} and G^{13}) 9
4.0 NMR of Assembly Process 9
5.0 X-Ray Diffraction and Scattering 10
6.0 Small-Angle Neutron Scattering (SANS) 10
6.1 Scattering Length Densities (SLDs) 10
6.2 Power Law Model 11
6.3 Flexible Elliptical Cylinder Models 12
6.4 Concentration Effects 14
7.0 Spin-Echo Small-Angle Neutron Scattering (SESANS) 15
8.0 Molecular Dynamics Simulations 16
9.0 Rheology 18
9.1 Pure Gels 18
9.1.1 Frequency Sweep 18

9.1.2 Amplitude Sweep 18
9.1.3 H_2O vs D_2O Time Sweep 20
9.2 Multicomponent Gels (with components 1 and 2) 20
9.2.1 Frequency Sweep 20
9.2.2 Amplitude Sweep 21
9.2 Varying Ratio Gels 21
10 Determination of the Degree of Association 21
11 Supplementary References 22

1.0. Experimantal Details

The data that support the findings of this study are available from the corresponding authors upon reasonable request.

1.1 Chemical List

Chemicals were obtained from the following suppliers: trifluoracetic acid (TFA), 3-aminobenzoic acid – **Fluorochem**, hexamethylenetetramine (HMTA), glucono-δ-lactone (GdL) – **Alfa Aesar**, phloroglucinol, 4-aminobenzoic acid, sodium deuteroxide (NaOD) – **Sigma Aldrich**, 3-amino-5-(trifluoromethyl)benzoic acid – **Apollo Scientific**, deuterium oxide (D_2O), d_6-dimethylsulfoxide (d_6-DMSO) – **Cambridge Isotope Laboratories**, dichloromethane (DCM), methylated spirits, sodium hydroxide (NaOH), hydrochloric acid (HCl) – **Fischer Chemical**.

1.2 NMR

NMR spectra were recorded on a Bruker AV 300 operating at 300 MHz. Chemical shifts (δ in ppm) were referenced to tetramethylsilane (TMS). *J* values are given in Hz and s, d, t, m are abbreviations corresponding to singlet, doublet, triplet, multiplet, respectively.

1.3 IR

IR spectra were recorded on Perkin-Elmer Spectrum 100 FT-IR Universal Sampling Accessory, deposited neat.

1.4 Mass Spectrometry

Atmospheric solids analysis probe (ASAP) mass spectrometry (MS) was recorded using a Shimadzu LCMS-2020 mass spectrometer.

1.5 pH Meter

The pH meter used was a Mettler Toledo SevenCompact, a two-point calibration was performed prior to measurement.

1.6 X-Ray Diffraction (XRD) and Scattering

XRD patterns were collected at room temperature using a Bruker D8 Discover equipped with a Lynxeye XE detector operating in reflection mode. Samples were dried at room temperature under vacuum filtration on filter paper. The material was scratched off the paper and run on a 2.5 cm diameter zero diffraction silicon holder during data collection. A blank of the zero diffraction holder was collected as a background data set and subtracted from the sample data. Data are plotted as arbitrary units as the intensity vs Q, the norm for scattering data using X-Rays.

1.7 Rheology

Rheological experiments were performed on a Bohlin nano II rheometer. A 40 mm aluminium cone at 4° was used with an operating gap of 300 μm gap and a solvent trap at 20°C. Gelator solution was immediately injected after addition of GdL into the operating gap to form contact with the cone and plate during gelation. **Time sweep** experiments were commenced immediately after injection of gelator solution. A torque of 0.02 μNm was applied at a frequency of 0.5 Hz, data was taken every 18 seconds. **Frequency sweeps** were performed immediately after the time sweep. A torque of 0.02 μNm was applied with a frequency range of 0.01 Hz to 100 Hz as a log scale. **Amplitude sweep** experiments were recorded after the frequency sweep at a frequency of 0.5 Hz.

1.8 Molecular Dynamic (MD) Simulations

All simulations were run using GROMACS 2021 release. Molecular topologies for each of the three gelator molecules were generated using the Automated Topology Builder web server.[1] Five gelator molecules were added to a cubic simulation box of side length 7.8 nm. SPC water was

added to a density of approximately 1000 g/L. This corresponds to approximately 15000 water molecules for each simulated gelator system. The box size and number of gelators was sufficient to give a concentration of approximately 1 %(w/w) of the gelators. The mixed water plus gelator systems were energy minimised using a conjugate gradients algorithm and then subjected to short consecutive 100 ps simulations in the NVT and NPT ensembles (with position restraints on the gelator molecules) to allow equilibration of temperature and pressure. The position restraints were removed and a production run of 100 ns in the NPT ensemble (300 K, 1 bar) was carried out for each gelator system, sampling the coordinates every 200ps. The following simulation parameters were used -

- LINCS constraints algorithm for bonded interactions of all atoms
- Electrostatic interactions calculated using the particle mesh Ewald summation method with a cutoff of 1.0 nm for both coulombic and van der Waals interactions
- v-rescale thermostat for temperature control to 300K and Berendsen barostat for pressure control to 1 bar (isotropic)
- GROMOS 53a6 force field

The radius of gyration of the gelator aggregate was analysed along the principal axes using the gmx gyrate command.

1.9 Small-Angle Neutron Scattering (SANS)

SANS measurements were carried out on the SANS2D time of flight (TOF) instrument at the ISIS Spallation Neutron Source, Rutherford Appleton Laboratory, Oxfordshire, UK (DOI of the experiment: 10.5286/ISIS.E.RB2010573). The scattered intensity was monitored at fixed sample to detector distance of 4 m. A polychromatic incident neutron beam with wavelengths from 1.75 to 16.5 Å, in TOF mode, generated a typical Q range of 0.00416 to 0.72191 Å^{-1}.[2]

Gelator solution was transferred to 1 mm Hellma cells immediately after the addition of GdL and the cells were mounted on an automatic sample changer at room temperature. The size of the beam at the sample position was set to 8 mm and the measurement times were approximately 40 minutes. The 2D raw scattering data were radially averaged, corrected for the sample transmission and efficiency of detector response. The instrumental and sample background scattering were subtracted using an empty cell. The data reduction was performed using Mantid software.[3] The differential scattering cross-section ($d\Sigma/d\Omega(Q)$ or $I(Q)$), in absolute units of cm^{-1} was obtained by calibration with the expected scattering from a partially deuterated polystyrene in accordance with established procedures.[4] The reduced data were then fitted using SasView software (version 4.2.2, http://www.sasview.org/).

1.10 Spin-Echo SANS (SESANS)

SESANS measurements were performed on the Larmor beamline at the ISIS Neutron and Muon Source (Rutherford Appleton Laboratory, Didcot, UK). Larmor is a time-of-flight SESANS instrument, which performs measurements in a magnetic field of a fixed strength (B) and inclination with respect to the neutron beam (θ_B) but with a range of wavelengths (λ) to vary the spin-echo length (Z) in a single measurement. Given the instrument configuration for this experiment, the wavelength range selected ($2 \leq \lambda \leq 12$ Å), and the magnet angle used (-30°), SESANS data were obtained over a length scale range of 0.9 to 13.8 µm. The samples were illuminated with a 4 mm square beam.

Gels were prepared and loaded into cells with a 10 mm path length. The samples were left to set before loading them into the instrument. Each SESANS measurement was ~15 min in duration, and empty instrument polarisations were measured once an hour. This was to ensure that the instrument was stable and to allow any outlying measurements to be excluded. Each sample polarisation (P) and empty beam polarisation (P_0) were measured approximately six times, and these were averaged to give greater signal-to-noise.

Data were processed in Mantid software[3] using bespoke routines to convert raw measurements of P and P_0 to the normalised SESANS signal, $\ln(P/P_0)/(t\lambda^2)$.

2.0 Synthesis and Characterisation

2.1 2,4,6-Triformylphloroglucio1

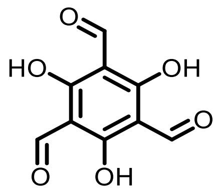

Figure S1. Molecular structure of 1,3,5-triformylphloroglucinol.

Synthesised following previously published procedure.[5, 6]

^{1}H NMR (d_6-DMSO, J/Hz, δ/ppm): 10.78 (s, 3H, CHO).

2.2 G_{111} (conformers C_3 and C_s 3:1)

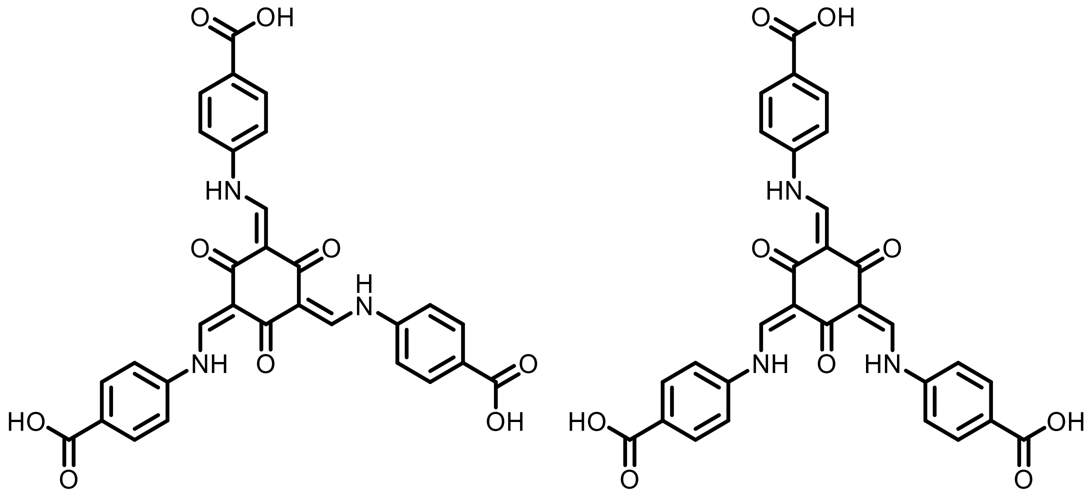

Figure S2. Molecular structure of $\mathbf{G_{111}}$ (C_3 left and C_s right).

Synthesised following previously published procedure.[5, 7]

^{1}H NMR (d_6-DMSO, J/Hz, δ/ppm): 13.32 (d, 3H, J=7.72, =CNH), 12.87 (dd, 2H, J=13.21, 8.40, =CNH), 8.76 (d, J=11.11, 5H, HC-N), 7.99 (m, 10H, Ar-H), 7.58 (m, 10H, Ar-H).

2.3 G_{222} (conformers C_3 and C_s 3:1)

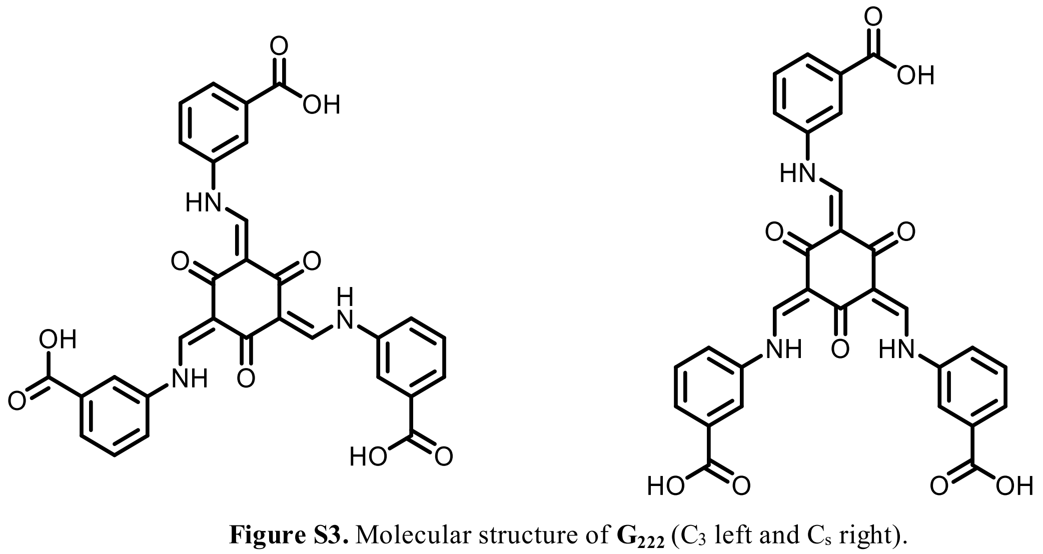

Figure S3. Molecular structure of $\mathbf{G_{222}}$ (C_3 left and C_s right).

3-Aminobenzoic acid (506 mg, 3.69 mmol) was added to a suspension of 2,4,6-triformylphloroglucinol (250 mg, 1.19 mmol) in methylated spirits (40 mL). The suspension was heated to reflux for 20 hours with stirring, after which the reaction was left to cool to room temperature and subsequently 0 °C. The crude product was collected by filtration, washed with deionised water (20 mL) and hot methylated spirits (100 mL). The final product was filtered and dried in an oven at 50°C. The synthesis is analogous to the literature procedures for the synthesis of **G**$_{111}$ and **G**$_{333}$.[5]

^{1}H NMR (d$_6$-DMSO, J/Hz, δ/ppm): 13.30 (m, 3H, =CNH), 12.90 (m, 2H, =CNH), 8.73 (d, J= 13.02, 3H, HC-N), 7.94 (m, 5H, Ar-H), 7.78 (m, 10H, Ar-H), 7.59 (m, 5H, Ar-H). **IR (cm^{-1})** 3070, 2634, 2360, 2342, 1709, 1615, 1575, 1448, 1449, 1425, 1234, 1193, 1116, 1039, 985, 923, 835, 668. **HRMS (m/z):** [M - H]$^-$ Calcd 566.12; Found 566.12.

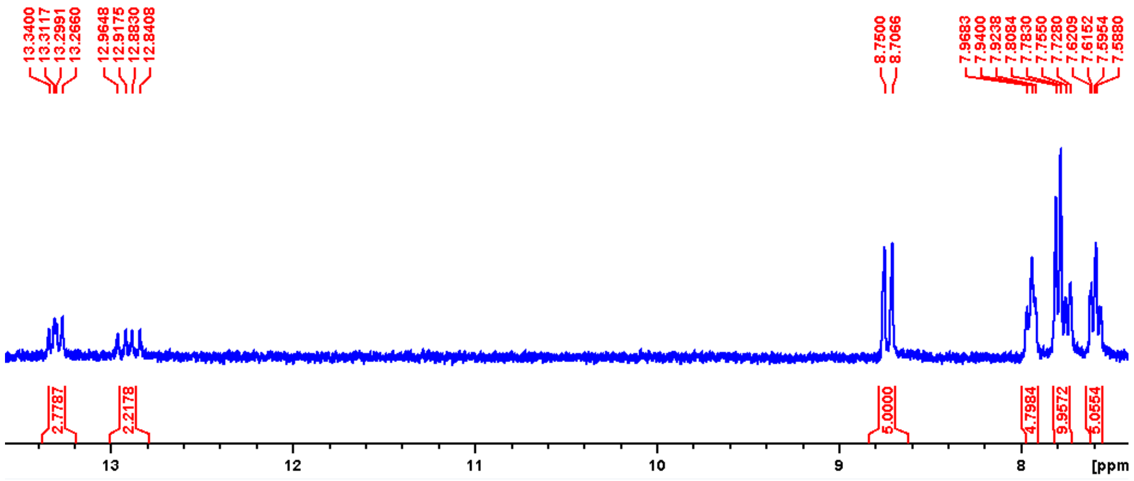

Figure S4. ^{1}H NMR spectrum of G$_{222}$.

2.4 G$_{333}$ (conformers C$_3$ and C$_s$ 3:1)

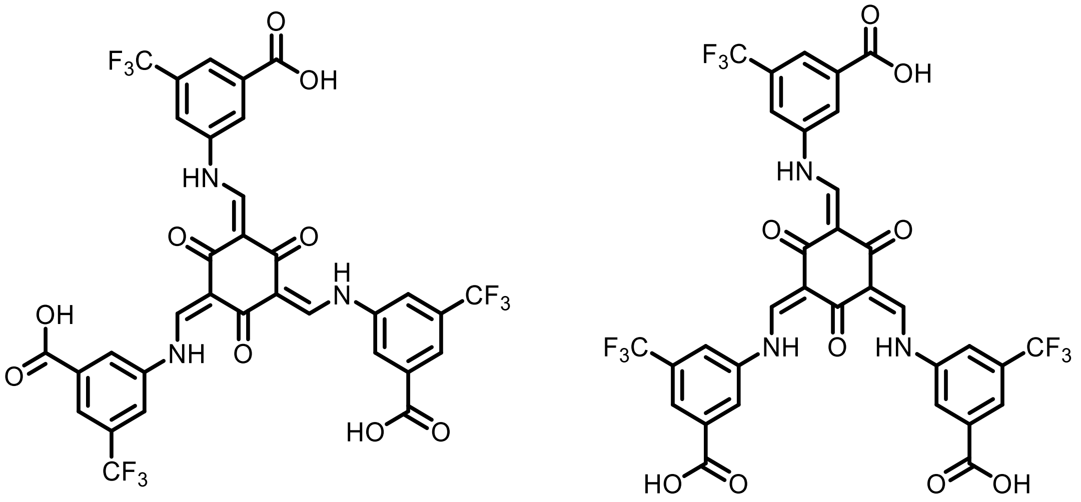

Figure S5. Molecular structure of G$_{333}$ (C$_3$ left and C$_s$ right).

Synthesised following previously published procedure.[5, 7]

^{1}H NMR (d$_6$-DMSO, J/Hz, δ/ppm): 13.24 (m, 3H, =CNH), 12.97 (m, 2H, =CNH), 8.74 (m, 5H, HC-N), 8.06 (m, 15, Ar-H).

2.5 pK_a Values

Table S1. pK_a values for starting materials and LMWGs.

**Compound**	**pK_a**
Triformylphloroglucinol	6.5*
4-Aminobenzoic Acid	4.9 (COOH)[8, 9], 2.4 (NH_2)[10] or 4.2*
3-Aminobenzoic Acid	3.1(COOH), 4.8 (NH_2)[10] or 4.2*
3-Amino-5-(trifluoromethyl)benzoic acid	3.6*
**G_{111}**	6.1-5.8[5]
**G_{222}**	6.5-5.9
**G_{333}**	5.2-5.0[5]

*pKa values calculated using ChemDraw Professional 22.2.0.3300.

2.5.1 pH Titration for G_{222}

To determine the apparent pK_a of gelator **G_{222}** a titration was performed. **G_{222}** (10 mg) was dissolved in 0.05 M NaOH (8 mL) and 1 M HCl was added in aliquots. The pH was recorded after each addition.

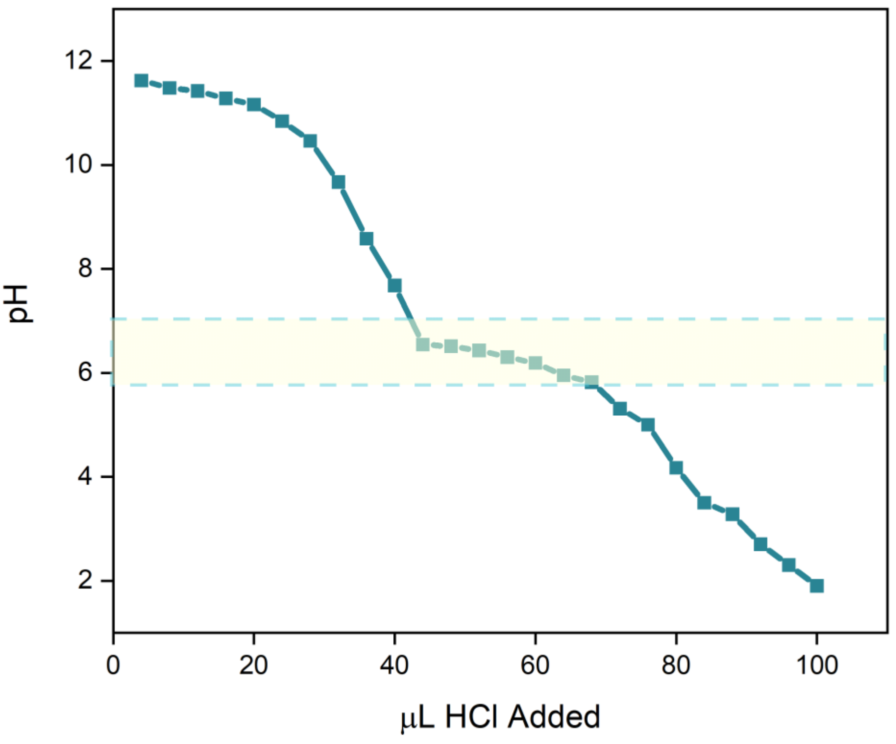

Figure S6. pH Titration for **G_{222}**.

3.0 Hydrogel Preparation

3.1 Single Component Gels (G_{111}, G_{222} and G_{333})

Gelator (7 mg) was dissolved in a pre-prepared NaOD/D_2O solution (0.5 M, 0.7 mL) and GdL (75 mg) was added.

3.2 Multicomponent Gels Route A ($G_{111}G_{222}$, and $G_{111}G_{333}$)

Gelator A (**G_{111}**) (3.5 mg) and gelator B (**G_{222}** or **G_{333}**) (3.5 mg or equivalent) were dissolved in a pre-prepared NaOD/D_2O solution (0.5 M, 0.7 mL) and GdL (75 mg) was added. Chemical analyses of these samples have shown that the components do not undergo dynamic covalent chemistry due to the tautomerisation.[5,7]

3.3 Multicomponent Gels Route B (G^{12} and G^{13})

Aminobenzoic acid A (**1**) (1.5 eq), aminobenzoic acid B (**2** or **3**) (1.5 eq) and 2,4,6-triformylphloroglucinol (1 eq) were dissolved in a pre-prepared NaOD/D_2O solution (0.5 M, 0.7 mL, 1 wt %) and the resulting solution was stirred at room temperature for 24 hours. GdL (75 mg) was then added.

4.0 NMR of Assembly Process

G_{111} (5 mg) and **G_{222}** (5 mg) were dissolved in D_2O/NaOD (0.075 M) and MeOH (5 μL) was added as an internal standard. GdL (30 mg) was added, and spectra were recorded every 10 minutes on a Bruker AV 400 NMR operating at 400.1 MHz.

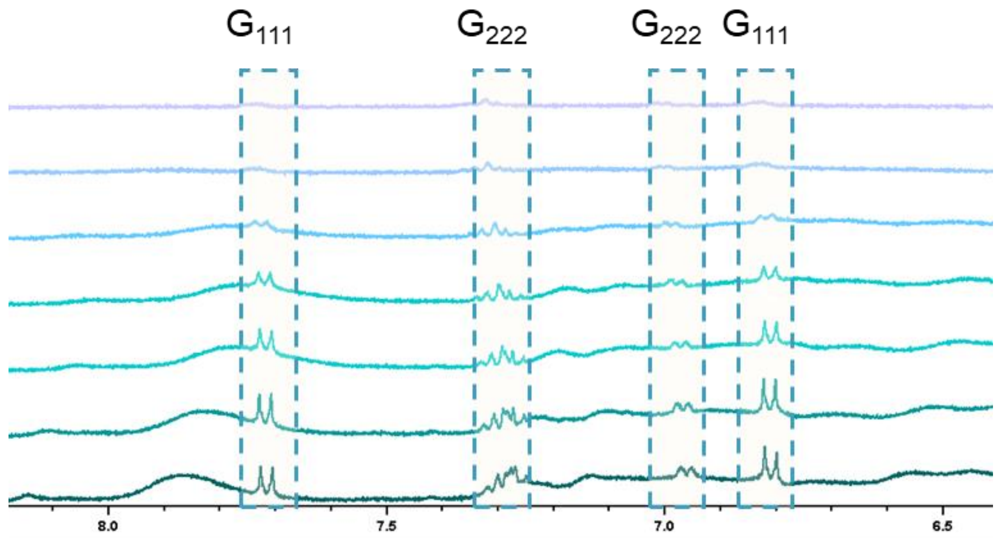

Figure S7. ^{1}H NMR traces of a **G_{111}** and **G_{222}** mixture were recorded every 10 minutes showing a simultaneous reduction in peak intensity over time.

5.0 X-Ray Diffraction and Scattering

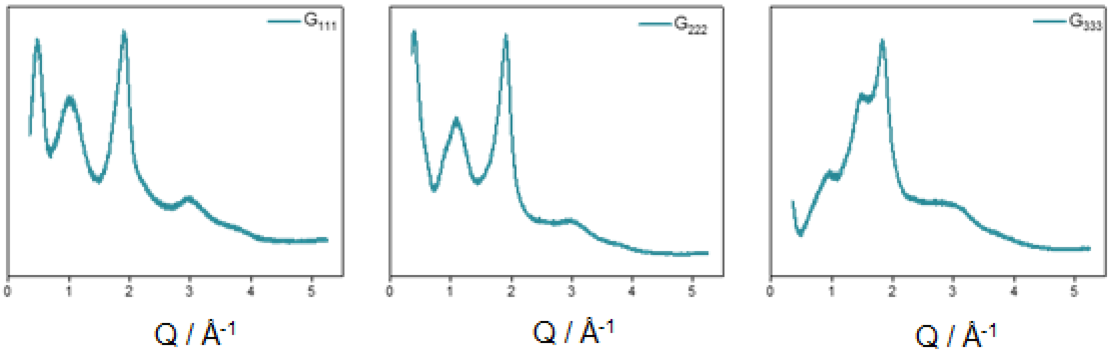

(a) (b) (c)

Figure S8. XRD patterns for $\mathbf{G_{111}}$, $\mathbf{G_{222}}$ and $\mathbf{G_{333}}$, from left to right as indicated. Y axis represents intensity in arbitrary units.

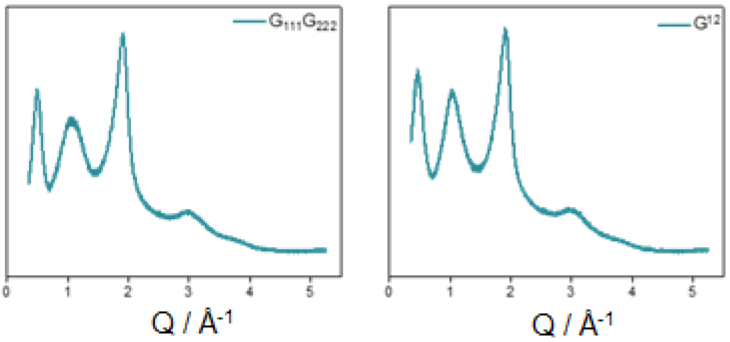

Figure S9. XRD patterns for $\mathbf{G_{111}G_{222}}$ (left) and G^{12} (right). Y axis represents intensity in arbitrary units.

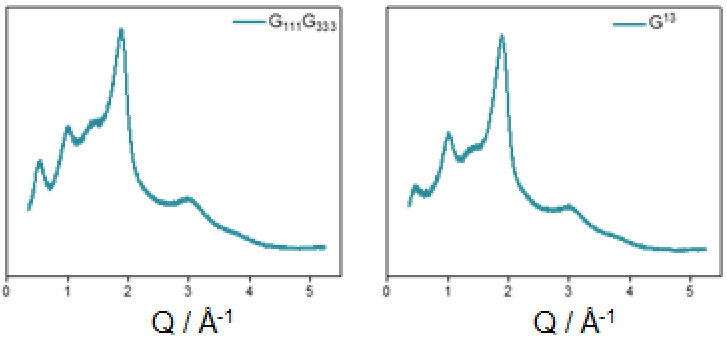

Figure S10. XRD patterns for $\mathbf{G_{111}G_{333}}$ (left) and $\mathbf{G^{13}}$ (right). Y axis represents intensity in arbitrary units.

6.0 Small-Angle Neutron Scattering (SANS)

6.1 Scattering Length Densities (SLDs)

All scattering length densities (SLDs), ρ (cm^{-2}), were calculated using software available from NIST.[11] The following equation was used:

$$\rho = \frac{N_A \rho_{bulk} \sum b_i}{M} \quad (1)$$

where N_A is Avogadro's number, ρ_{bulk} is the density of the sample, assumed to be 1 g cm^{-3}, M is the molar mass and $\sum b_i$ represents the sum of scattering length of all atoms in the molecule. SLD values are listed in Table S2.

Table S2. SLD values for the pure gelators.

Compound	SLD /Å^{-2}
$\mathbf{G_{111}}$	2.14 x 10^{-6}
$\mathbf{G_{222}}$	2.14 x 10^{-6}
$\mathbf{G_{333}}$	2.21 x 10^{-6}

6.2 Power Law Model

To determine the Q dependence, the SANS data were fitted to a power law:

$$I(Q) = I(0) \times Q^{-\alpha} + B \quad (2)$$

where $I(0)$ is the scale, α characterises the Q dependence and B represents the background. Fitting parameters are reported in Table S3. SANS data and fits are shown in Figures S11 to S13. As indicated in Figures S11 to S13, the SANS data were not fitted using the entire Q range available. This is to improve the quality of fits by neglecting data with larger deviations at the lowest and highest Q values (see Figure S11, for example).

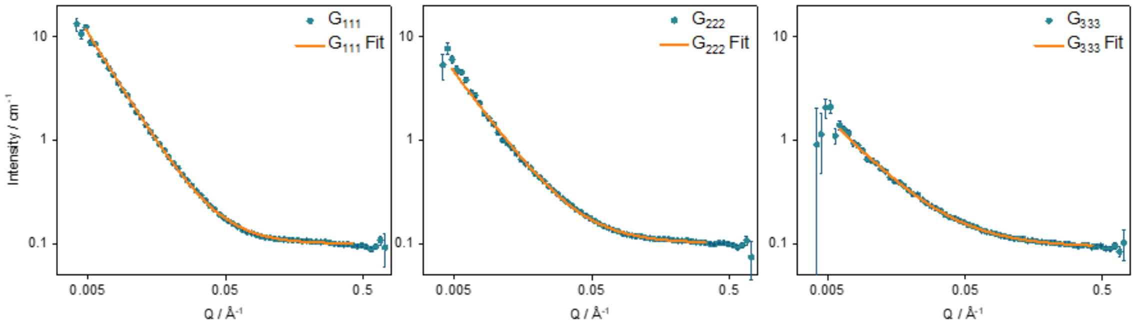

Figure S11. SANS data (full circles) for pure gels $\mathbf{G_{111}}$, $\mathbf{G_{222}}$ and $\mathbf{G_{333}}$ (from left to right). The lines are fits, of not all the data, using the power law model.

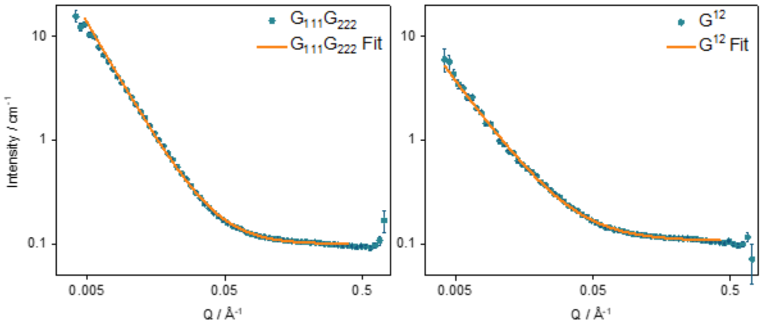

Figure S12. SANS data (full circles) for gels $\mathbf{G_{111}G_{222}}$ (left) and $\mathbf{G^{12}}$ (right). The lines are fits, of not all the data, using the power law model.

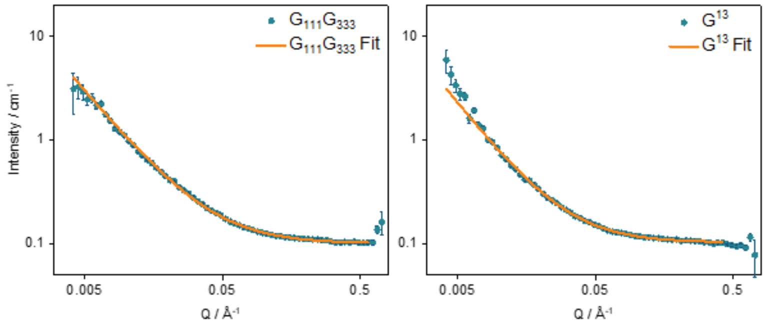

Figure S13. SANS data (full circles) for gels $\mathbf{G_{111}G_{333}}$ (left) and $\mathbf{G^{13}}$ (right). The lines are fits, of not all the data, using the power law model.

Table S3. Fitting parameters as extracted from fits to the SANS data using the power law model, equation (2), for all gels.

Gel	***I*(0) / 10^{-4} cm^{-1}**	***B* / 10^{-1} cm^{-1}**	**α**	**χ^2**
$\mathbf{G_{111}}$	1.29 ± 0.03	1.001 ± 0.002	2.125 ± 0.006	4.24
$\mathbf{G_{222}}$	3.25 ± 0.10	1.014 ± 0.003	1.788 ± 0.008	3.48
$\mathbf{G_{333}}$	10.3 ± 0.4	0.931 ± 0.003	1.38 ± 0.01	1.50
$\mathbf{G_{111}G_{222}}$	0.867 ± 0.020	0.100 ± 0.002	2.241 ± 0.006	4.65
$\mathbf{G^{12}}$	3.19 ± 0.11	1.075 ± 0.003	1.752 ± 0.009	2.79
$\mathbf{G_{111}G_{333}}$	7.81 ± 0.24	1.008 ± 0.003	1.544 ± 0.008	2.46
$\mathbf{G^{13}}$	3.49 ± 0.16	1.031 ± 0.003	1.64 ± 0.01	2.03

6.3 Flexible Elliptical Cylinder Models

Several models were tested during fitting including the cylinder, flexible cylinder (as used in literature for supramolecular gel materials[12, 13]), and the stacked disk model (mimicking the face-to-face stacking of our discotic LMWGs). Fits over the entire Q range were poor with χ^2 upwards of 15. This is unsurprising given the Q dependency is close to 2. The elliptical cylinder model was also tested, and this was similarly unsuccessful but with the addition of the Kuhn length parameter, the flexible elliptical cylinder model provided good fits for all SANS data of our gel samples.

The form factor given by the model describes a flexible cylinder with an elliptical cross-section, as shown in Figure S14.

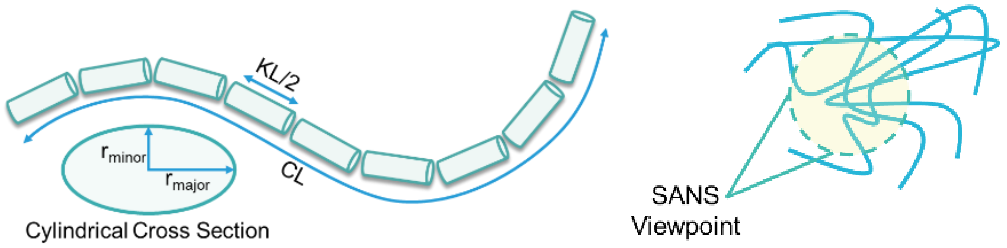

Figure S14. Schematic representation of the flexible elliptical cylinder model and the viewpoint provided by SANS of the gel network.

Each cylinder may be considered as a chain of locally stiff segments, the length of which is equivalent to half the Kuhn length (KL). The contour length (CL) is the total length of the chain and therefore the greater the difference between the contour and Kuhn length, the more flexible the cylinder. Gel networks which constitute gels are multilevel structures with fibril overlaps and

entanglements and therefore the contour length here can be thought of as the network ‘mesh size’ rather than the length of an individual cylinder (fibre).

The elliptical cross-section has a minor radius (r_{minor}) and a major radius (r_{major}). The major radius/minor radius is returned from the model as the axis ratio, which must be greater than one. The larger the value, the more elliptical the cross-section.

Our data is likely the sum of three contributions: a flat background dominating at high Q, scattering from individual fibres in the intermediate range and further scattering contributions at low Q. As the contour length is outside the size accessible with the measured Q range (no plateau at low Q) it was fixed at 25,000 Å for gel with $\mathbf{G_{111}}$ and $\mathbf{G_{222}}$ and 2,500 for $\mathbf{G_{333}}$, in order to provide the best fit.

Table S4. Values extracted from the Flexible Elliptical Cylinder model for all gels.

Gel	Scale / 10^{-5} cm^{-1}	Background / 10^{-1} cm^{-1}	Kuhn Length /Å	Radius / Å	Axis Ratio	χ^2
$\mathbf{G_{111}}$	8.4 ± 0.3	1.002 ± 0.003	281 ± 41	8.5 ± 0.1	14 ± 2	4.9
$\mathbf{G_{222}}$	33.3 ± 0.1	1.055 ± 0.003	40 ± 5	7.4 ± 0.4	2.80 ± 0.01	2.6
$\mathbf{G_{333}}$	45.0 ± 0.3	1.000 ± 0.003	146 ± 6	8.50 ± 0.03	2.4 ± 0.1	3.5
$\mathbf{G_{111}G_{222}}$	9.65± 0.01	1.010 ± 0.002	144 ± 8	7.9 ± 0.5	12 ± 2	3.5
$\mathbf{G^{12}}$	38.8 ± 0.4	1.114 ± 0.003	45 ± 6	7.60 ± 0.02	2.5 ± 0.2	1.9
$\mathbf{G_{111}G_{333}}$	41.5± 0.2	1.091 ± 0.003	97 ± 4	8.5 ±0.3	3.1 ± 0.1	3.5
$\mathbf{G^{13}}$	37.0 ± 0.3	1.056 ± 0.003	88 ± 3	8.47 ± 0.03	2.5 ± 0.1	3.6

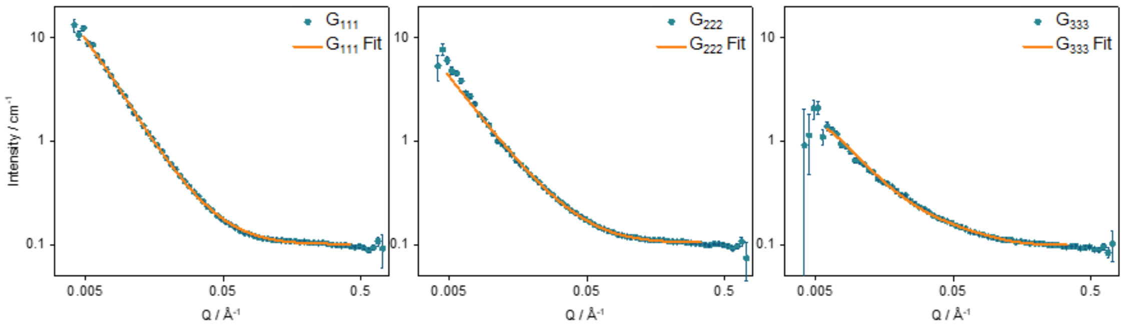

Figure S15. SANS data (full circles) for pure gels $\mathbf{G_{111}}$, $\mathbf{G_{222}}$ and $\mathbf{G_{333}}$ from left to right and fits using the flexible elliptical cylinder model in SasView.

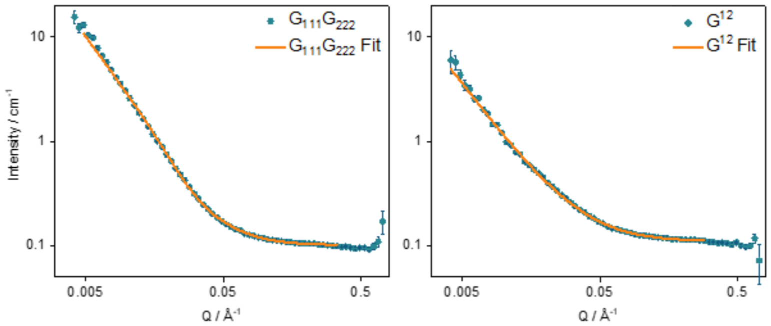

Figure S16. SANS data (full circles) for gels $\mathbf{G_{111}G_{222}}$ (left) and $\mathbf{G^{12}}$ (right) and fits using the flexible elliptical cylinder model in SasView.

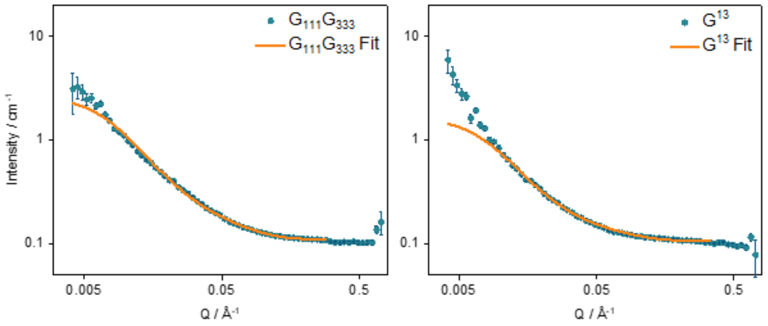

Figure S17. SANS data (full circles) for gels $\mathbf{G_{111}G_{333}}$ (left) and $\mathbf{G^{13}}$ (right) and fits using the flexible elliptical cylinder model in SasView.

6.4 Concentration Effects

A comparison between the SANS data of the pure gels, $\mathbf{G_{111}}$ and $\mathbf{G_{222}}$, is given in Figure S18. The comparison between data at 1wt% and 5wt% indicates that structural changes take place, with increasing concentration. This is a well known phenomenon observed in polymer solutions and attributed to overlap between polymer coils.

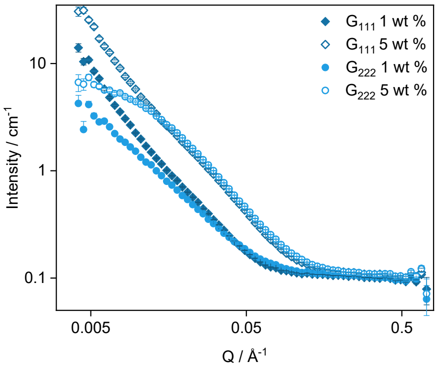

Figure S18. SANS data for $\mathbf{G_{111}}$ and $\mathbf{G_{222}}$ at different concentrations, as indicated.

The $\mathbf{G_{111}}$ SANS data at 2 wt% and 5 wt% cannot be fitted using the flexible elliptical cylinder model in SasView.[14] This is shown in Figure S19 where calculated curves using fitting parameters obtained from the $\mathbf{G_{111}}$ 1 wt% data (Table S4) are compared to the experimental data, at higher concentration. To account for differences in the scattered intensities as a result of increased concentration, the scale and background B were increased to fit the 2 and 5 wt% curves. Deviations could be accounted for by including a Lorentzian component to extract a correlation length (not shown in Figure S19).

The shoulder at high Q is indicative of structural changes. These changes are much more pronounced for $\mathbf{G_{222}}$ (Figure S18 and S20). The $\mathbf{G_{222}}$ SANS data at this concentration can be fitted using the correlation length model in SasView:

$$I(Q) = \frac{A}{Q^n} + \frac{C}{1 + (Q\xi)^m} + B \tag{3}$$

where the first term is a power law and the second term is a Lorentzian function which is often used to model the scattering from polymer chains, at relatively high concentrations. A and C are scale parameters indicating the relative contributions of the two components. To fit the data, we fixed the exponent n to 1.8 (to equal the α value in Table S3 obtained for $\mathbf{G_{222}}$). We obtain a correlation length of 65 Å and a power exponent m = 2.3. We attribute changes in the scattering

behaviour of the gels to the formation of a network structure at high concentration. This is consistent with the relatively short correlation length extracted from the fits.

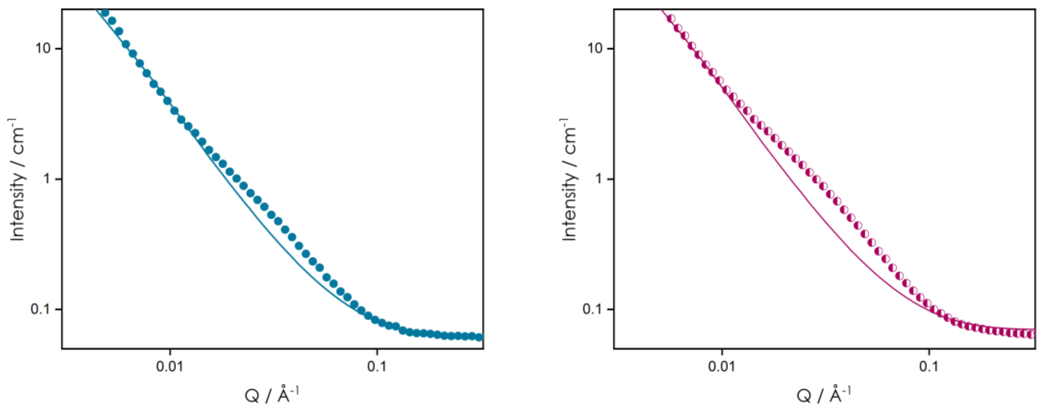

Figure S19. SANS data for $\mathbf{G_{111}}$ at two different concentrations: 2 wt% (left) and 5 wt% (right). The lines are calculated curves using fitting parameters obtained from the elliptical flexible cylinder model for $\mathbf{G_{111}}$ at a lower concentration i.e. 1 wt%. To account for concentration differences, the scale and background were adjusted to fit the 2 and 5 wt% curves. Deviations could be accounted for by including a Lorentzian component to extract a correlation length.

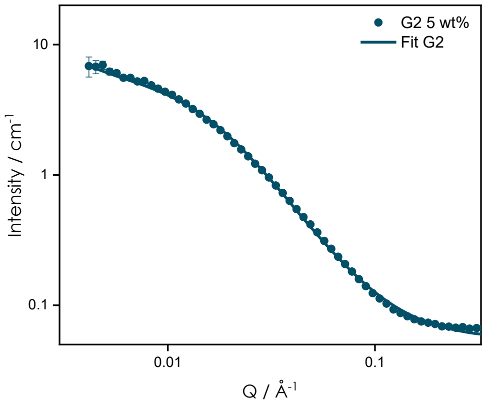

Figure S20. SANS data for $\mathbf{G_{222}}$ at 5 wt%. The line represents a fit, of not all the data, to the data using the correlation length model in SasView.

7.0 Spin-Echo Small-Angle Neutron Scattering (SESANS)

Detailed discussions of SESANS data and analysis can be found in the literature.[15] Analysis of the SESANS data in this study are simplified because the normalised SESANS signals have reached saturation level, being constant for all spin-echo length (Z) values. We will refer to this parameter as P_∞/P_0. This parameter can easily be converted into the total scattering (Σ_t), the average number of times a neutron scatters when traversing a sample, by the expression $P_\infty/P_0=\exp(-\Sigma_t)$.[15]

The normalised SESANS signal can be related to the correlation length (ξ) by the following expression:

$$\frac{ln(P_\infty/P_0)}{t\lambda^2} = -\xi\Delta\rho^2\phi(1-\phi) \quad (4)$$

where $\Delta\rho^2$ is the contrast which is given by the difference in scattering length density between the particles and solvent, and ϕ is the volume fraction of the particles.[15]

It is only possible to use the above expression to analyse the value of P_∞/P_0 for homogenous particles, but this assumption is valid for the systems studied here.[16] As can be seen from equation (3), if the contrast ($\Delta\rho^2$) and the volume fraction (ϕ) are the same, then there is a direct proportionality between the normalised SESANS signal and the correlation length.

The normalised SESANS signal was found to be constant as a function of spin echo length, for all our samples (Figure 5b and c). This indicates that there are no structural features at the length scales probed (between approximately 0.9 and 13.8 μm). In this case, the saturation value should be constant and we show this in Figures 5b and c using a straight line.

8.0 Molecular Dynamics Simulations

Snapshot conformations of the final self-assembled configuration after 100ns of the three gelator systems are shown in Figures S31-S33. All gelators show a stacked conformation. $\mathbf{G_{111}}$ and $\mathbf{G_{222}}$ exhibited the most ordered stacking, whilst for $\mathbf{G_{333}}$ a greater degree of disorder was seen for each of three separate simulations. In these a single $\mathbf{G_{333}}$ gelator was observed to insert itself into a stack of the four other gelators in a conformation that disrupted the regularity of the self-association. Repeat simulations (x3 in total) for the $\mathbf{G_{333}}$ gelator also showed a partially disordered aggregate structure.

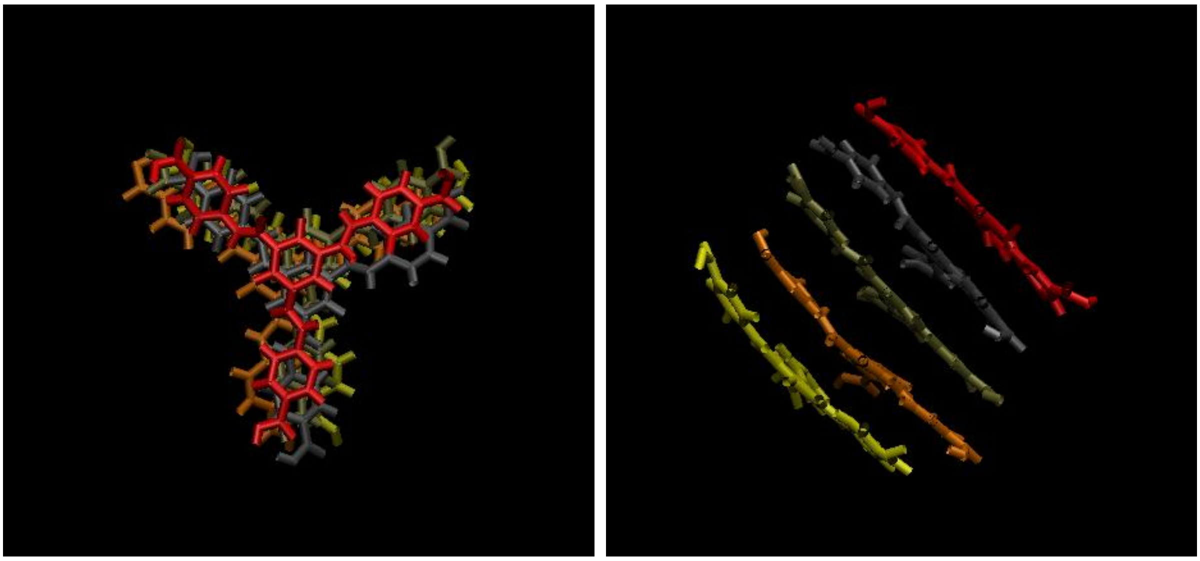

Figure S21. Snapshot conformation of the aggregate of five $\mathbf{G_{111}}$ gelators after 100ns of MD simulation.

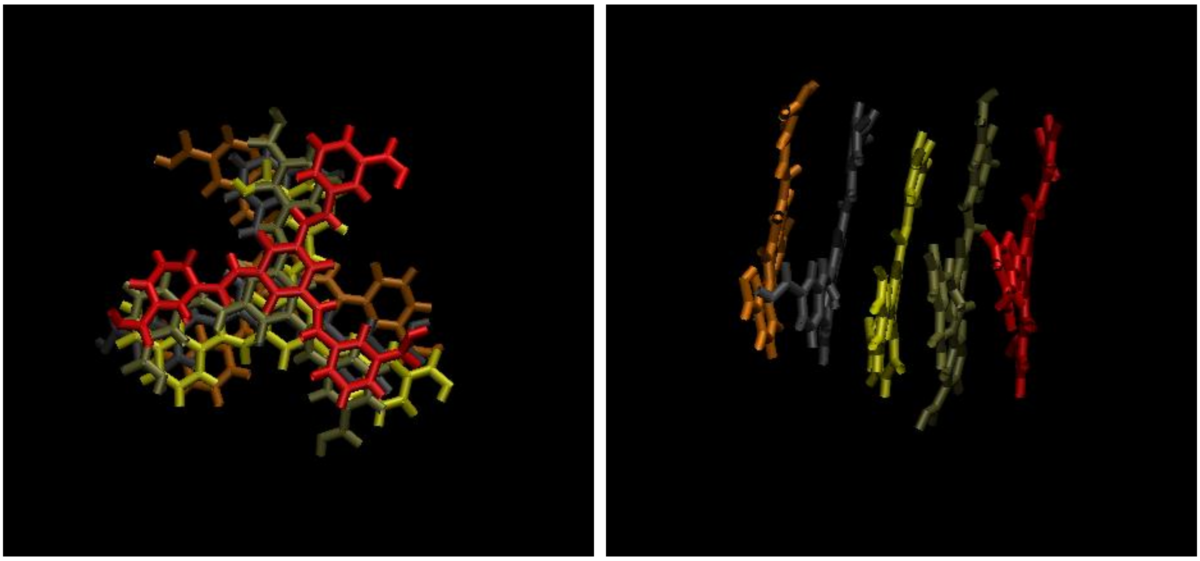

Figure S22. Snapshot conformation of the aggregate of five $\mathbf{G_{222}}$ gelators after 100ns of MD simulation.

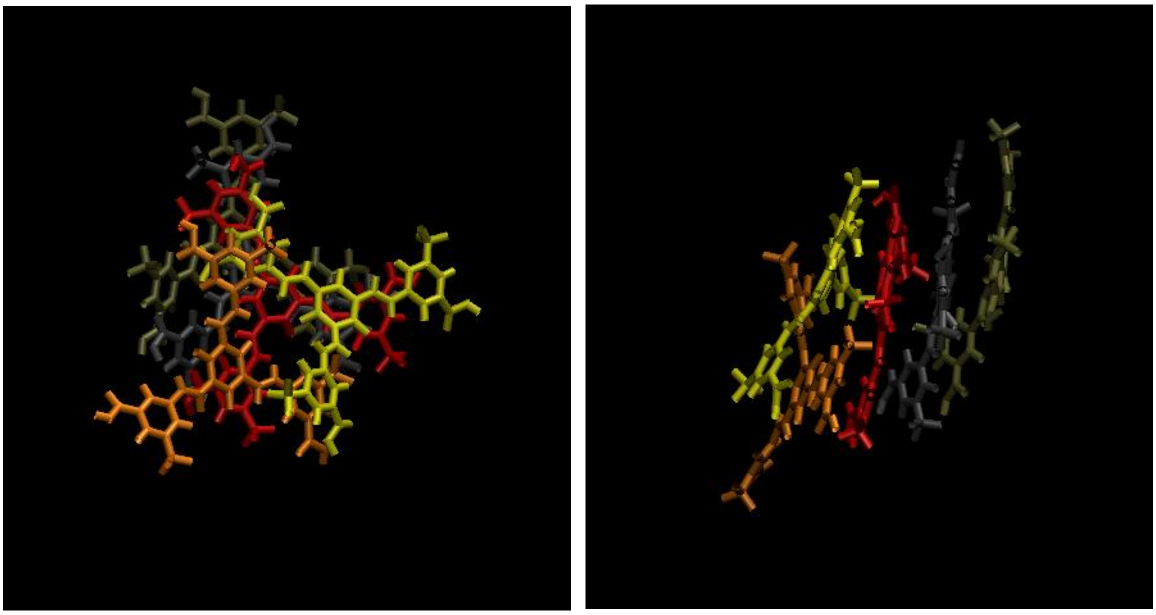

Figure S23. Snapshot conformation of the aggregate of five $\mathbf{G_{333}}$ gelators after 100ns of MD simulation.

The dimensions of the self-assembled aggregates were calculated as the radius of gyration along the three principal axes of the cluster. The results are shown in Table S5. Here the shortest dimension (x-axis) corresponds to the axis down the centre of the aggregates, and y and z directions across the aggregate. To convert the radius of gyration to an apparent radius, we used the methodology proposed by Bogusz et al.[16] for micelles. Here, they define an effective radius for micelles which is based on the relationship between the radius of a solid sphere (R_S) and its radius of gyration (R_g) where,

$$\boldsymbol{R_S} = \sqrt{\frac{\mathbf{5}}{\mathbf{3}}}.\boldsymbol{R_g} \tag{5}$$

Table S5 – Average components of the radius of gyration and effective radii calculated along the principal axes of the gelator aggregates.

**Gelator**	**Average components of radius of gyration along principal axes (Å)**			**Effective radii along principal axes (Å)**		
	$\mathbf{R_{gx}}$	$\mathbf{R_{gy}}$	$\mathbf{R_{gz}}$	**x**	**y**	**z**
$\mathbf{G_{111}}$	6.4 ± 0.2	7.1 ± 0.1	7.4 ± 0.1	8.3 ± 0.3	9.2 ± 0.1	9.6 ± 0.3
$\mathbf{G_{222}}$	6.2 ± 0.1	6.8 ± 0.2	7.1 ± 0.2	8.0 ± 0.2	8.8 ± 0.2	9.2 ± 0.3
$\mathbf{G_{333}}$	6.3 ± 0.1	7.3 ± 0.1	8.2 ± 0.1	8.2 ± 0.1	9.5 ± 0.2	10.6 ± 0.2

The calculated radii of the gelator aggregates compare favourably to those determined from the SANS data.

9.0 Rheology

9.1 Pure Gels

9.1.1 Frequency Sweep

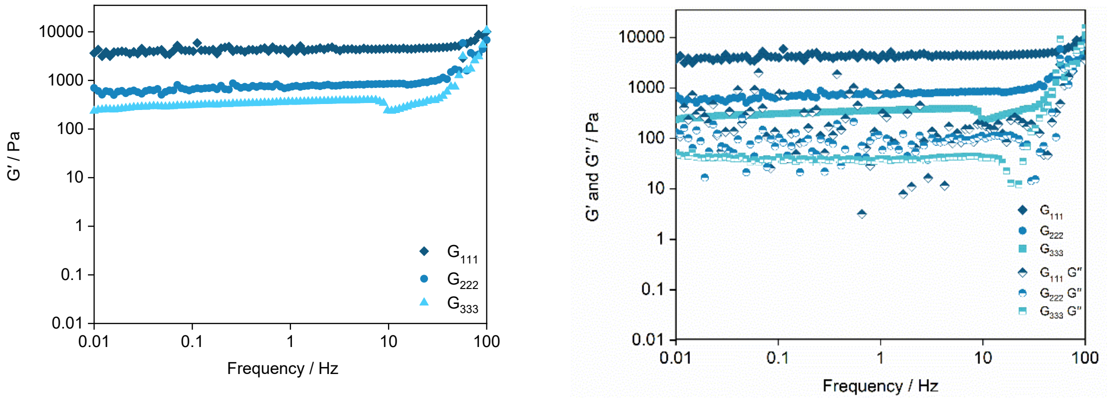

Figure S24. Frequency sweep measurements for pure gels $\mathbf{G_{111}}$, $\mathbf{G_{222}}$ and $\mathbf{G_{333}}$. Left is G′ plotted only while on the right both G′ and G″ are plotted.

9.1.2 Amplitude Sweep

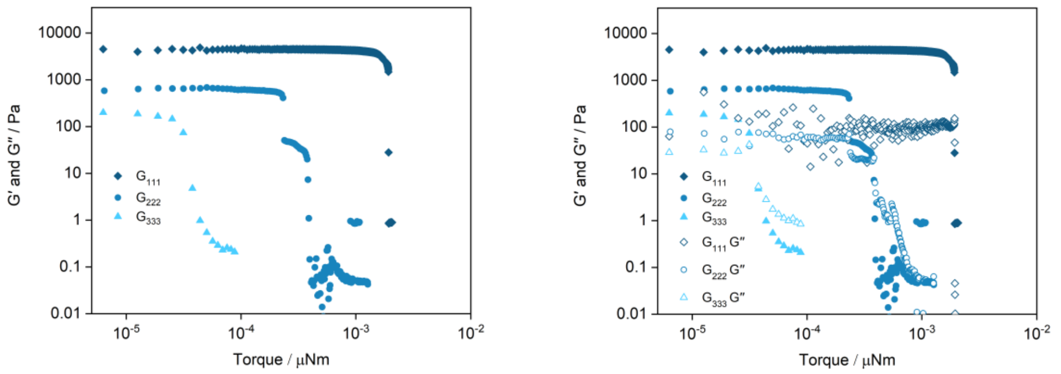

Figure S25. Amplitude sweep measurements for pure gels $\mathbf{G_{111}}$, $\mathbf{G_{222}}$ and $\mathbf{G_{333}}$. Left is G′ plotted only while on the right both G′ and G″ are plotted.

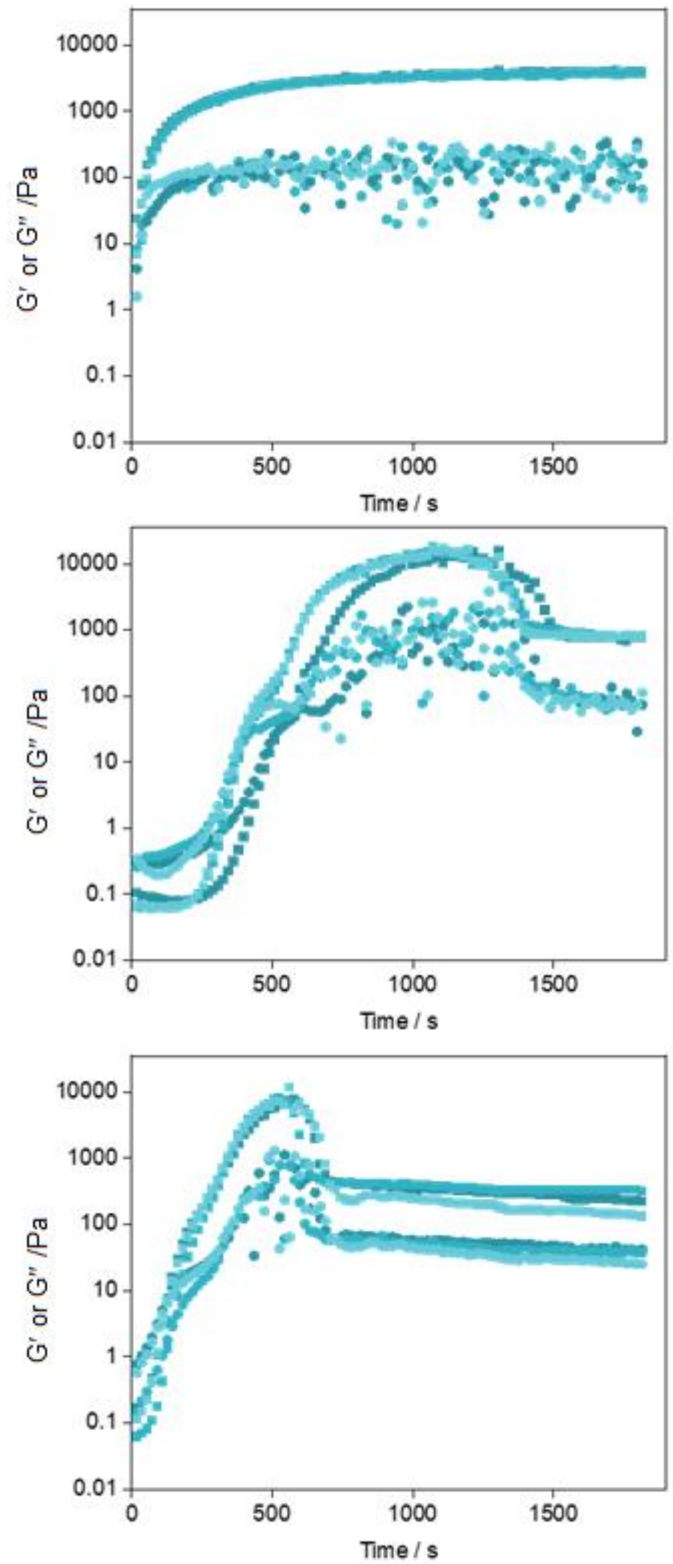

Figure S26. Time sweeps showing changes in G′ (squares) and G″ (circles) for pure gels. For top to bottom: (a) $\mathbf{G_{111}}$, (b) $\mathbf{G_{222}}$ and (c) $\mathbf{G_{333}}$. Measurements were repeated three times.

9.1.3 H_2O vs D_2O Time Sweep

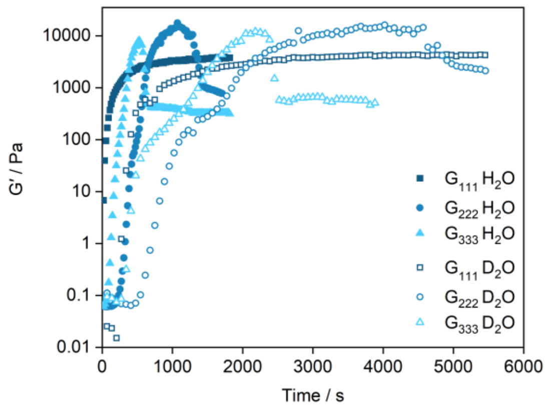

Figure S27. Time sweep measurement for pure gels $\mathbf{G_{111}}$, $\mathbf{G_{222}}$ and $\mathbf{G_{333}}$ in H_2O (solid fill), and D_2O (outline). G′ plotted only to make the information imparted by the data clearer. The plot indicates that the final gels for D_2O and H_2O are similar rheologically even though the kinetics are different. G′ and G″ data can be found in Fig. S26.

9.2 Multicomponent Gels (with components 1 and 2)

9.2.1 Frequency Sweep

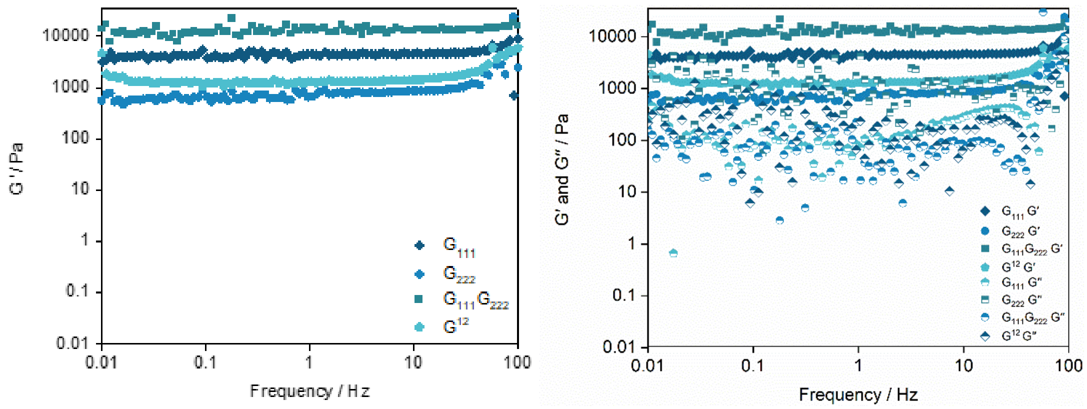

Figure S28. Frequency sweep measurements for gels $\mathbf{G_{111}}$, $\mathbf{G_{222}}$, $\mathbf{G_{111}G_{222}}$ and $\mathbf{G^{12}}$. Left is G′ data plotted only while on the right both G′ and G″ are plotted.

9.2.2 Amplitude Sweep

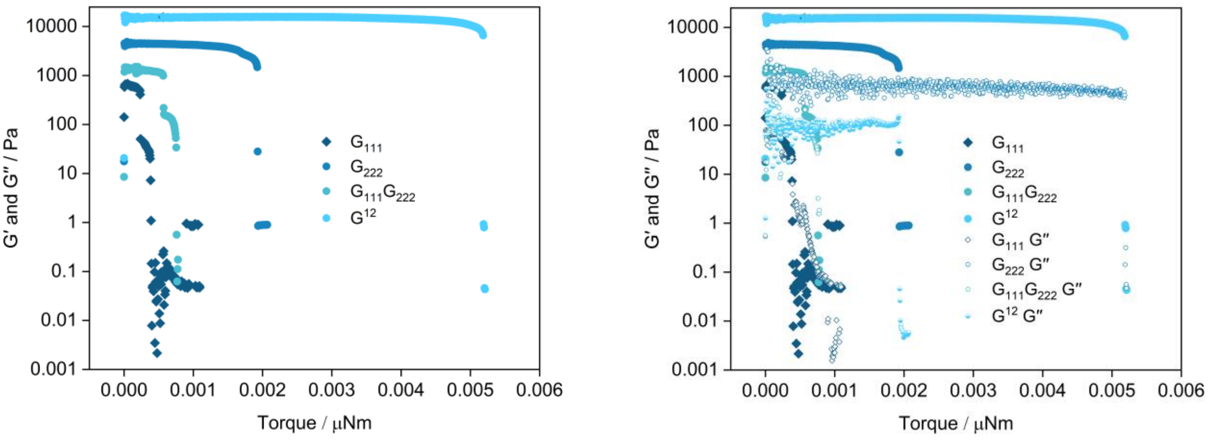

Figure S29. Amplitude sweep measurements for gels $\mathbf{G_{111}}$, $\mathbf{G_{222}}$, $\mathbf{G_{111}G_{222}}$ and $\mathbf{G^{12}}$. On the left only G′ data plotted. On the right both G′ and G″ data are plotted.

9.2 Varying Ratio Gels

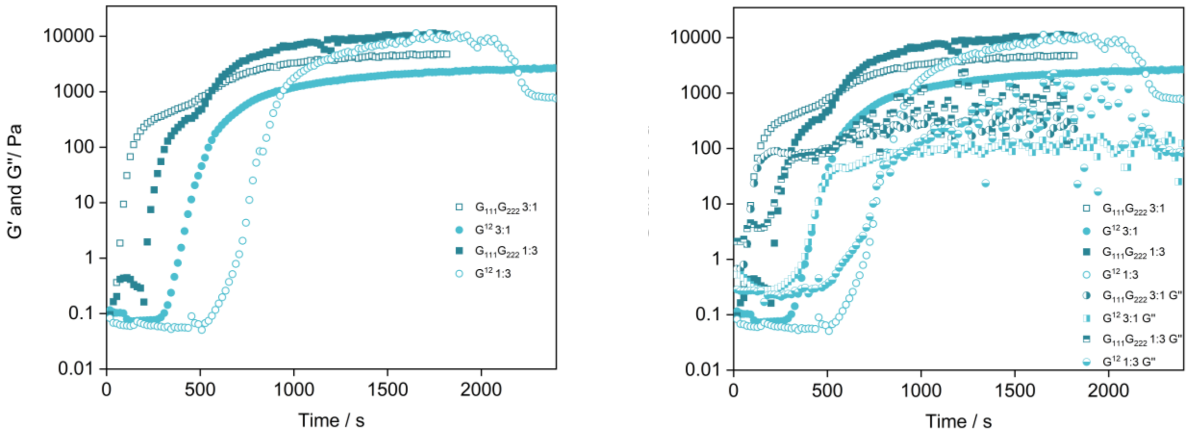

Figure S30. Time sweep measurements for gels $\mathbf{G_{111}G_{222}}$ and $\mathbf{G^{12}}$ at different ratio of LMWGs. Left is G′ data only while on the right both G′ and G″ are plotted.

10 Determination of the Degree of Association

We can estimate the degree of association of the fibres from the SANS data and fitting parameters obtained using the flexible elliptical cylinder model. The radii and axial ratios extracted from the fits are given in Table S4. From these, we can calculate the area of the fibre cross section which is elliptical. The LMWGs can be assumed to be spherical with an average radius equal to ca. 8 Å, in line with MD simulations, giving an upper estimate of the degree of association as reported in Table S6.

Table S6 – Cross sectional área of the fibres from SANS data analysis and degree of association.

Gelator	Area cross section /Å^2	Degree of Association
**G_{111}**	3178	15.8
**G_{222}**	495	2.5
**G_{333}**	544	2.7

11 Supplementary References

1. Malde, A. K.; Zuo, L.; Breeze, M.; Stroet, M.; Poger, D.; Nair, P. C.; Oostenbrink, C.; Mark, A. E., An Automated Force Field Topology Builder (ATB) and Repository: Version 1.0. *Journal of Chemical Theory and Computation* **2011,** *7* (12), 4026-4037.
2. Jackson, A. J., Introduction to small-angle neutron scattering and neutron reflectometry. *NIST Center for Neutron Research* **2008**, 1-24.
3. Arnold, O.; Bilheux, J. C.; Borreguero, J. M.; Buts, A.; Campbell, S. I.; Chapon, L.; Doucet, M.; Draper, N.; Ferraz Leal, R.; Gigg, M. A.; Lynch, V. E.; Markvardsen, A.; Mikkelson, D. J.; Mikkelson, R. L.; Miller, R.; Palmen, K.; Parker, P.; Passos, G.; Perring, T. G.; Peterson, P. F.; Ren, S.; Reuter, M. A.; Savici, A. T.; Taylor, J. W.; Taylor, R. J.; Tolchenov, R.; Zhou, W.; Zikovsky, J., Mantid—Data analysis and visualization package for neutron scattering and μSR experiments. *Nuclear Instruments and Methods in Physics Research Section A: Accelerators, Spectrometers, Detectors and Associated Equipment* **2014,** *764*, 156-166.
4. Wignall, G. D.; Bates, F. S., Absolute calibration of small-angle neutron scattering data. *Journal of Applied Crystallography* **1987,** *20* (1), 28-40.
5. Foster, J. S.; Prentice, A. W.; Forgan, R. S.; Paterson, M. J.; Lloyd, G. O., Targetable Mechanical Properties by Switching between Self-Sorting and Co-assembly with *In Situ* Formed Tripodal Ketoenamine Supramolecular Hydrogels. *ChemNanoMat* **2018,** *4* (8), 853-859.
6. Chong, J. H.; Sauer, M.; Patrick, B. O.; MacLachlan, M. J., Highly Stable Keto-Enamine Salicylideneanilines. *Organic Letters* **2003,** *5* (21), 3823-3826.
7. Foster, J. S.; Żurek, J. M.; Almeida, N. M. S.; Hendriksen, W. E.; Le Sage, V. A. A.; Lakshminarayanan, V.; Thompson, A. L.; Banerjee, R.; Eelkema, R.; Mulvana, H.; Paterson, M. J.; Van Esch, J. H.; Lloyd, G. O., Gelation Landscape Engineering Using a Multi-Reaction Supramolecular Hydrogelator System. *Journal of the American Chemical Society* **2015,** *137* (45), 14236-14239.
8. International Union of, P.; Applied, C.; Serjeant, E. P.; Dempsey, B.; International Union of, P.; Applied Chemistry Commission on Electrochemical, D., *Ionisation constants of organic acids in aqueous solution*. Pergamon Press: Oxford, 1979.
9. Settimo, L.; Bellman, K.; Knegtel, R. M. A., Comparison of the Accuracy of Experimental and Predicted pKa Values of Basic and Acidic Compounds. *Pharmaceutical Research* **2014,** *31* (4), 1082-1095.
10. CRC Handbook of Chemistry and Physics. **2016**.
11. Kienzle, P. Scattering Length Density Calculator http://www.ncnr.nist.gov/resources/activation/.
12. Guilbaud, J.-B.; Saiani, A., Using small angle scattering (SAS) to structurally characterise peptide and protein self-assembled materials. *Chem. Soc. Rev.* **2011,** *40* (3), 1200-1210.
13. Willemen, H. M.; Marcelis, A. T. M.; Sudhölter, E. J. R.; Bouwman, W. G.; Demé, B.; Terech, P., A Small-Angle Neutron Scattering Study of Cholic Acid-Based Organogel Systems. *Langmuir* **2004,** *20* (6), 2075-2080.
14. Doucet, M.; Cho, J. H.; Alina, G.; Attala, Z. ; Bakker, J.; Bouwman, W.; Butler, P.; Campbell, K.; Cooper-Benun, T.; Durniak, C.; Forster, L.; Gonzales, M.; Heenan, R.; Jackson,

A.; King, S.; Kienzle, P.; Krzywon, J.; Nielsen, T.; O'Driscoll, L.; Potrzebowski, W.; Prescott, S.; Ferraz Leal, R.; Rozycko, P.; Snow, T.; Washington, A. *SasView version 5.0.3*, 2020. http://www.sasview.org/.
15. Andersson, R.; Heijkamp, L.; Schepper, I.; Bouwman, W., Analysis of spin-echo small-angle neutron scattering measurements. *J. Appl. Crystallogr.* **2008,** *41*, 868-885.
16. Washington, A. L.; Li, X.; Schofield, A. B.; Hong, K.; Fitzsimmons, M. R.; Dalgliesh, R.; Pynn, R., Inter-particle correlations in a hard-sphere colloidal suspension with polymer additives investigated by Spin Echo Small Angle Neutron Scattering (SESANS). *Soft Matter* **2014,** *10* (17), 3016-3026.
